# Physical and cognitive impact following SARS-CoV-2 infection in a large population-based case-control study

**DOI:** 10.1038/s43856-023-00326-5

**Published:** 2023-07-06

**Authors:** Hilma Holm, Erna V. Ivarsdottir, Thorhildur Olafsdottir, Rosa Thorolfsdottir, Elias Eythorsson, Kristjan Norland, Rosa Gisladottir, Gudrun Jonsdottir, Unnur Unnsteinsdottir, Kristin E. Sveinsdottir, Benedikt A. Jonsson, Margret Andresdottir, David O. Arnar, Asgeir O. Arnthorsson, Kolbrún Birgisdottir, Kristbjorg Bjarnadottir, Solveig Bjarnadottir, Gyda Bjornsdottir, Gudmundur Einarsson, Berglind Eiriksdottir, Elisabet Eir Gardarsdottir, Thorarinn Gislason, Magnus Gottfredsson, Steinunn Gudmundsdottir, Julius Gudmundsson, Kristbjorg Gunnarsdottir, Anna Helgadottir, Dadi Helgason, Ingibjorg Hinriksdottir, Ragnar F. Ingvarsson, Sigga S. Jonasdottir, Ingileif Jonsdottir, Tekla H. Karlsdottir, Anna M. Kristinsdottir, Sigurdur Yngvi Kristinsson, Steinunn Kristjansdottir, Thorvardur J. Love, Dora Ludviksdottir, Gisli Masson, Gudmundur Norddahl, Thorunn Olafsdottir, Isleifur Olafsson, Thorunn Rafnar, Hrafnhildur L. Runolfsdottir, Jona Saemundsdottir, Svanur Sigurbjornsson, Kristin Sigurdardottir, Engilbert Sigurdsson, Martin I. Sigurdsson, Emil L. Sigurdsson, Valgerdur Steinthorsdottir, Gardar Sveinbjornsson, Emil A. Thorarensen, Bjarni Thorbjornsson, Brynja Thorsteinsdottir, Vinicius Tragante, Magnus O. Ulfarsson, Hreinn Stefansson, Thorsteinn Gislason, Mar Kristjansson, Runolfur Palsson, Patrick Sulem, Unnur Thorsteinsdottir, Gudmundur Thorgeirsson, Daniel F. Gudbjartsson, Kari Stefansson

**Affiliations:** 1 deCODE genetics/Amgen Inc., Reykjavik, Iceland; 2 Internal Medicine and Emergency Services, Landspitali—The National University Hospital of Iceland, Reykjavik, Iceland; 3 School of Humanities, University of Iceland, Reykjavik, Iceland; 4 Faculty of Medicine, School of Health Sciences, University of Iceland, Reykjavik, Iceland; 5 National Institute of Hearing and Speech in Iceland, Reykjavik, Iceland; 6 Clinical Laboratory Services, Diagnostics and Blood Bank, Landspitali—The National University Hospital of Iceland, Reykjavik, Iceland; 7 Mental Health Services, Landspitali—The National University Hospital of Iceland, Reykjavik, Iceland; 8 Perioperative Services, Landspitali—The National University Hospital of Iceland, Reykjavik, Iceland; 9 Department of Family Medicine, University of Iceland, Reykjavik, Iceland; 10 Development Centre for Primary Health Care in Iceland, Reykjavik, Iceland; 11 Faculty of Electrical and Computer Engineering, University of Iceland, Reykjavik, Iceland; 12 School of Engineering and Natural Sciences, University of Iceland, Reykjavik, Iceland

**Keywords:** Diseases, Medical research, Viral infection

## Abstract

**Background:**

Persistent symptoms are common after SARS-CoV-2 infection but correlation with objective measures is unclear.

**Methods:**

We invited all 3098 adults who tested SARS-CoV-2 positive in Iceland before October 2020 to the deCODE Health Study. We compared multiple symptoms and physical measures between 1706 Icelanders with confirmed prior infection (cases) who participated, and 619 contemporary and 13,779 historical controls. Cases participated in the study 5–18 months after infection.

**Results:**

Here we report that 41 of 88 symptoms are associated with prior infection, most significantly disturbed smell and taste, memory disturbance, and dyspnea. Measured objectively, cases had poorer smell and taste results, less grip strength, and poorer memory recall. Differences in grip strength and memory recall were small. No other objective measure associated with prior infection including heart rate, blood pressure, postural orthostatic tachycardia, oxygen saturation, exercise tolerance, hearing, and traditional inflammatory, cardiac, liver, and kidney blood biomarkers. There was no evidence of more anxiety or depression among cases. We estimate the prevalence of long Covid to be 7% at a median of 8 months after infection.

**Conclusions:**

We confirm that diverse symptoms are common months after SARS-CoV-2 infection but find few differences between cases and controls in objective parameters measured. These discrepancies between symptoms and physical measures suggest a more complicated contribution to symptoms related to prior infection than is captured with conventional tests. Traditional clinical assessment is not expected to be particularly informative in relating symptoms to a past SARS-CoV-2 infection.

## Plain language summary

Persistent symptoms are commonly reported after SARS-CoV-2 infection, and this is often described as long Covid. We compared different symptoms reported following SARS-CoV- 2 infection with the results obtained during various medical evaluations that are often used to assess health, such as blood tests, smell tests, taste tests, hearing tests, etc. We compared symptoms and test results between 1,706 Icelanders who had been infected previously with SARS-CoV-2 infection (cases) and 14,398 individuals who had not been infected (controls). Out of 88 assessed symptoms, 41 were more common in cases than controls. However, relatively few differences were seen in the results obtained from the various medical evaluations (cases had poorer smell and taste test results, slightly less grip strength, and slightly poorer memory recall than controls). The differences seen between symptoms and results of medical evaluations suggests that conventional clinical tests may not be informative in relating symptoms to a past SARS-CoV-2 infection.

## Abstract (web summary)

Holm, Ivarsdottir, Olafsdottir et al. compare symptoms and physical measures between Icelanders post SARS-CoV-2 infection with uninfected controls. From reported symptoms, they estimate the prevalence of long COVID as 7% at a median of 8 months after infection, while objective differences between cases and controls in the physical measures were few.

## Introduction

Severe acute respiratory syndrome coronavirus 2 (SARS-CoV-2), which causes coronavirus disease 2019 (Covid-19), emerged in December 2019^1^. As of January 2023, the pandemic has resulted in 664 million confirmed cases worldwide^2^ while the true number of infected persons is likely much higher^3^.

Covid-19 is an acute respiratory infection with the potential for widespread extrapulmonary complications^4,5^. Several studies on the sequelae of Covid-19 in hospitalized patients found persistent symptoms and multiorgan abnormalities^6–8^, but good physical and functional recovery was reported for most severe Covid-19 survivors one year after the infection^9^. Evidence of various long-term effects of the SARS-CoV-2 infection on physical and mental health in those with milder disease, the majority of infected persons, is emerging. Protracted post-acute infection symptoms (long Covid)^10^, including fatigue, dyspnea, and brain fog have been described, with prevalence estimates ranging from 3% to 52%^11–13^ in non-hospitalized cohorts. However, a comprehensive comparison of post-Covid symptoms and objective measures in non-hospitalized persons is lacking.

To investigate health outcomes after SARS-CoV-2 infection, we invited all Icelanders who had tested positive for the virus by qPCR^14^ or antibodies^15^ to the SARS-CoV-2 nucleocapsid (N) protein prior to October 2020 (cases), and a set of individuals who had been similarly tested but with negative results (contemporary controls), to participate in a modified deCODE Health Study (dHS)^16^, a prospective cohort study including questionnaires on symptoms and comprehensive physiological, cognitive and blood testing. History of prior infection was verified with measurement of anti-N antibodies^15^ at the time of the study visit (current vaccinations do not lead to the production of anti-N antibodies). Cases participated in the study 5–18 months after the acute infection. In addition to contemporary controls, our study includes a large control dataset of persons who participated in the dHS prior to the pandemic (historical controls), facilitating assessment of time effects, importantly the pandemic itself, on measures. Furthermore, a subset of cases participated twice in the dHS, before and after the infection, allowing for the assessment of longitudinal measures. Similar longitudinal measures were available for a subset of controls. Our study is mostly of non-hospitalized persons as only 5% of cases required hospitalization during the acute infection as was typical for SARS-CoV-2 infection prior to the vaccination era.

In our study, diverse symptoms were more commonly reported by those with prior SARS-CoV-2 infection than by controls, with 41 out of 88 assessed symptoms associating with past infection. However, few of the 150 objective physiological, cognitive, and blood parameters assessed differed between the two groups; cases had poorer smell and taste test results, less grip strength, and poorer memory recall than controls. Observed differences in grip strength and memory recall were small. We estimated the prevalence of long Covid to be 7%.

## Methods

### Ethical consideration

This study was approved by the Icelandic National Bioethics Committee (VSN-15-214 with amendments). Written informed consent was obtained from all participants, in accordance with the Declaration of Helsinki. Personal identifiers were encrypted by a third-party system overseen by the Icelandic Data Protection Authority^17^.

### Study design and participants

The dHS^16^ is a prospective cohort study in Iceland with extensive phenotypic and genotypic information produced and collected from the participants. More than 16,000 individuals participated in the study between its initiation in June 2016 and November 2021, aged between 18 and 97 years at recruitment. Participation in the study includes questionnaires about health and lifestyle, multiple physiological, cognitive, and blood tests, and authorization to access health-related information from registries and medical records, from where comprehensive information about comorbidities was obtained (Supplementary Methods).

The dHS was paused in March 2020 due to the pandemic. To study the health consequences of SARS-CoV-2 infection, a modified dHS, termed the dHS Covid Study, was launched in September 2020. Added measures in the dHS Covid Study included the C19Q questionnaire on both the presence and frequency of symptoms during the previous four weeks, designed by us. We also added these questionnaires to assess symptoms of anxiety, depression, stress, health anxiety, and fatigue: General Anxiety Disorder-7 (GAD-7^18^), Patient Health Questionnaire-9 (PHQ-9^19^), Perceived Stress Scale (PSS^20^), Short Health Anxiety Inventory (SHAI^21^), and Symptom Impact Questionnaire (SIQR^22^), respectively, as well as the Satisfaction With Life Scale (SWLS^23^) and 36-Item Short Form Survey (36-SF^24^) to assess health-related quality of life. We added tests of taste and orthostatic intolerance and made other changes described in Supplementary Methods.

As cases, we invited all Icelanders over 18 years of age, 3098 persons, who had tested positive for SARS-CoV-2 by qPCR or by antibodies against the viral nucleocapsid protein (anti-N) by October 2020, the information provided by the Directorate of Health, to participate in the dHS Covid Study five or more months after the acute infection. The majority of those infected (96%) had either been identified through targeted qPCR testing aimed at those at high risk for infection (mainly those who were symptomatic, had recently traveled to high-risk countries, or had contact with infected persons), or through population screening^14^. Others (4%) were identified through antibody testing^15^. Of note, at this time, the presence of anti-N antibodies indicated prior infection, not vaccination, as the nucleocapsid protein was not targeted by any approved vaccine. Of the 3098 presumed cases invited, 1777 participated in the study between September 2020 and November 2021 (Supplementary Figure 1). Levels of anti-N antibodies were measured at the time of the study and 88 persons were excluded from the case analysis based on anti-N antibodies being lower than 0.2 (Supplementary Figure 2) resulting in 1706 confirmed cases participating in the study (Supplementary Figure 1).

As contemporary controls, we invited 1017 persons from a pool of Icelanders who had undergone PCR testing and/or anti-N antibody measurement with negative results, information available to us from the Directorate of Health (Supplementary Figure 1). The invited controls were age and sex-matched to the invited cases but no other selection criteria were applied. We sent an invitation to 274 individuals between September and December 2020, 210 between December 2020 and February 2021, 308 between February 2021 and June 2021, and 221 between June 2021 and September 2021. Of the 1017 persons, 636 participated in the study between September 2020 and November 2021, and 619 (97.3%) were confirmed as contemporary controls by the absence of anti-N antibodies at the time of study participation, while 17 individuals were found to have anti-N antibodies levels over 1 (2.7%) and were therefore treated as cases in the analysis (Supplementary Figure 1). This resulted in 619 confirmed contemporary controls (Supplementary Figure 1). The small fraction of individuals with previous negative PCR tests that were found to have anti-N antibodies levels over 1 in our study shows a low false negative rate of PCR tests in our population. Of the 1706 cases, 125 persons had also participated in the dHS before the pandemic, allowing for a comparison of within-individual measures from before and after the infection (longitudinal measures).

As historical controls, we used information about 13,779 individuals who participated in the dHS before the pandemic, between June 2016 and March 2020 (Supplementary Figure 1). The total control group of the study is thus comprised of 14,398 persons, 13,779 who participated in the dHS before the pandemic, between June 2016 and March 2020 (historical controls) and 619 contemporary controls. Included in both numbers are 295 controls who participated twice in the dHS, before and during the pandemic.

In addition to being administered to all participants in the dHS Covid Study (1706 cases and 619 contemporary controls), an online version of the C19Q, assessing symptoms in the previous four weeks, was sent to a subset of the historical controls who had undergone PCR testing and/or anti-N antibody measurement with negative results, 2000 persons, age and sex-matched to the cases, and 760 responded, resulting in a C19Q control group of 1379 persons (Supplementary Figure 3).

For the GAD-7, PHQ-9, PSS, SWLS, and 36-SF questionnaires, we obtained additional data from the Icelandic iStopMM study^25^ for up to 264 cases and 20,459 contemporary controls for longitudinal assessment and for up to 33,099 other controls. In comparison of cases vs. all available controls for questionnaire scales, the data was restricted to individuals over 45 in accordance with the age distribution in the iStopMM data.

In this study, we report the relationship between the history of SARS-CoV-2 infection and results from the following measures and tests: (a) health and symptom questionnaires including C19Q, GAD-7, PHQ-9, PSS, SHAI, SIQR, SWLS, and 36-SF; (b) physiological measurements of height, weight, BMI, blood pressure, heart rate, oxygen saturation, body composition by whole-body dual-energy X-ray absorptiometry (DXA) scan, grip strength, smell test, taste test, hearing test, spirometry, cardiopulmonary exercise test, ambulatory sleep test; (c) cognitive tests: Digit Coding^26^, Letter and Category fluency^27^, Logical Memory^27,28^, Spatial Working Memory^27,29^, Trail Making Tests^27,30^, and Wechsler Abbreviated Scale of Intelligence^31,32^, and d) blood tests. The test procedures are described in Supplementary Methods.

### Severity of the acute SARS-CoV-2 infection

All persons diagnosed with SARS-CoV-2 infection by qPCR in Iceland were monitored by the Telehealth monitoring service (TMS)^33^ of the Covid-19 outpatient clinic at Landspitali—the National University Hospital in Reykjavik, Iceland. We scaled the severity of the acute infection from 0 (least severe) to 8 (most severe) based on three factors: (1) intensity of treatment, (2) severity assessment by the TMS, and 3) self-assessment (Supplementary Table 1).

### Long Covid symptom cluster

The term long Covid has been used to describe symptoms that develop during or following SARS-CoV-2 infection and last for more than four weeks per the National Institute for Health and Care Excellence guidelines^34^ or two months with an impact on daily function according to the World Health Organization (WHO) definition^35^. We assigned long Covid to cases who reported at least one of the following symptoms for five or more days per week: fatigue, lack of concentration, memory disturbance, dyspnea, or weakness, or at least one of the following for three or more days per week: malaise after physical exertion, chest pain, tachycardia in the four weeks prior to study visit, assessed with the C19Q, 5–18 months after infection. For comparison, we assessed how many of the controls fulfilled the same criteria.

### Statistics and reproducibility

With the C19Q we assessed both the presence and frequency of symptoms. For ease of interpretability, we treated the answers as binary traits for logistic regression (in general, absence/very infrequent symptom vs other), presenting ORs. As a robustness check, we also analyzed the answers as quantitative traits, yielding results similar to the results using logistic regression.

We tested all measures for association with SARS-CoV-2 infection adjusting for age and sex. Adjustment for comorbidities (obesity, hypertension, asthma, type 2 diabetes, cancer, and coronary artery disease) had minimal effect on associations and thus we report unadjusted results but show both in Supplementary Data. We applied two complementary study designs, allowing for mindful consideration of the trade-off between statistical power vs. screening for confounding effects when testing for association of SARS-CoV-2 and the numerous health-related traits. (A) We compared outcome measures of cases and all available control data. To account for time effects in (A), we tested for difference in measures between (i) cases and contemporary controls (restricting data to measures during the pandemic) and between ii) contemporary and historical controls where divergence from a null finding could indicate a time effect in A). We further plotted the data on physiological and blood traits against time of measure to explore batch effects. We observed batch effects for the hearing test, oxygen saturation, grip strength, and blood tests and for those traits measured data for controls was restricted to using more recent measures for historical controls (measures after 2017 for grip strength and after 2019 for hearing) or using only contemporary controls (oxygen saturation and blood tests, Supplementary Figure 4, Supplementary Methods). (B) We exploited a subset of the data that allows for a controlled before-and-after study, i.e., longitudinal measures for the same individuals collected before and during the pandemic (before and after infection for cases, with similar time duration between repeat measures for controls) providing the added benefit of accounting simultaneously for time effect and time-invariant individual heterogeneity, while acknowledging reduced power.

When testing for association between SARS-CoV-2 infection and other phenotypes, logistic regression was performed for binary traits, and linear regression was performed for quantitative traits. The physiological traits were regressed against SARS-CoV-2 status (1 for SARS-CoV-2 cases, 0 for controls), adjusting for age at time of measure and sex. The cognitive traits were similarly regressed against SARS-CoV-2 status, adjusting for age at time of measure, sex, and level of education obtained from the online questionnaire. Level of education was defined as a quantitative variable ranging from zero to six in the following manner: zero for no education, one for primary school, two for high school, three for other secondary education, four for an undergraduate university degree, five for Master’s degree and six for Doctorate degree. When testing for association between phenotypes and severity of the acute infection, the traits were regressed against the severity scale. *P* values for all regression analyses were obtained with a likelihood ratio test. Individuals with missing data were not used in association analyses. To test for sensitivity of the results to comorbidity, we tested for association between phenotypes and SARS-CoV-2 as described above with indicator variables added for obesity, coronary artery disease, type II diabetes, asthma, hypertension, and cancer, restricting the data to non-missing observations in the comorbidity indicators. When comparing measurements for individuals that participated in the dHS both before and during the pandemic, we subtracted pre-pandemic measures from those taken during the pandemic for both cases and controls and regressed the difference against SARS-CoV-2 status, adjusting for age, sex, and time between measures using linear regression.

For two of the cognitive traits, TMT-A and TMT-B, we log-transformed the test scores because of right-skewness in their distributions. Test scores for each of the 12 cognitive traits were then adjusted for age, gender, and education in a linear model prior to rank-based inverse normal transformation. The normalized and adjusted cognitive test scores were then used in association analyses to report effect estimates in SD units.

We calculated the frequency of memory impairment, defined as z-score of less than or equal to −1.5, among cases and controls for logical memory measures, adjusting for age, gender, and educational level. Since cases and controls are not perfectly matched on those three covariates, we estimated parameters for those covariates using the control data as training set, applying the corresponding, measure-specific, parameters to create z-scores for cases and controls.

To establish association with SARS-CoV-2 infection, we required (1) association when comparing cases with all available controls using the following thresholds accounting for multiple testing: *P* = 0.05/96 = 5 × 10^−4^ for health and symptom questionnaire data, *P* = 0.05/87 = 6 × 10^−4^ for physiological measures and cognitive tests, and *P* = 0.05/63 = 8 × 10^−4^ for blood tests, and (2) at least one of the following, as statistical power varies across measures: (i) consistent association results (same direction and non-heterogeneity in the effect estimates) with (1) when comparing cases with contemporary controls, (ii) consistent association results with (1) when comparing the subset of cases to the subset of controls with longitudinal measures. For symptoms and objective measures that associated with prior SARS-CoV-2 infection we required a *P* < 0.05 to establish a correlation with time from infection and the severity of the infection. For measures that did not associate with prior SARS-CoV-2 infection per se, we required the same multiple testing thresholds as listed above to establish an association with the severity of the infection. We used multiple linear or logistic regression for association testing. Analyses were performed in R, version 3.6.0.

### Patient and public involvement

No patients or members of the public were involved in the conceptualization or design of this study nor in the interpretation of the results.

### Reporting summary

Further information on research design is available in the Nature Portfolio Reporting Summary linked to this article.

## Results

### Study design and study participants

**Fig. 1:**
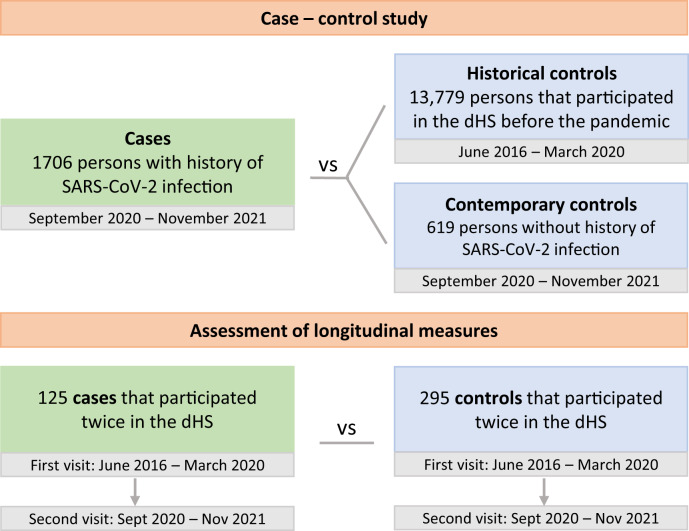
The dHS Covid study design. Cases and contemporary controls participated in the dHS Covid Study between September 2020 and November 2021. Historical dHS controls participated in the study between June 2016 and March 2020. Through the iStopMM study we obtained additional GAD-7, PHQ-9, PSS, SWLS, and 36-SF scores obtained both before and during the pandemic. To establish an association with prior SARS-CoV-2 infection, we required association when comparing cases with all available controls, accounting for multiple testing, and consistent results when comparing cases with contemporary controls or when comparing the subset of cases to the subset of controls with longitudinal measures.

**Fig. 2:**
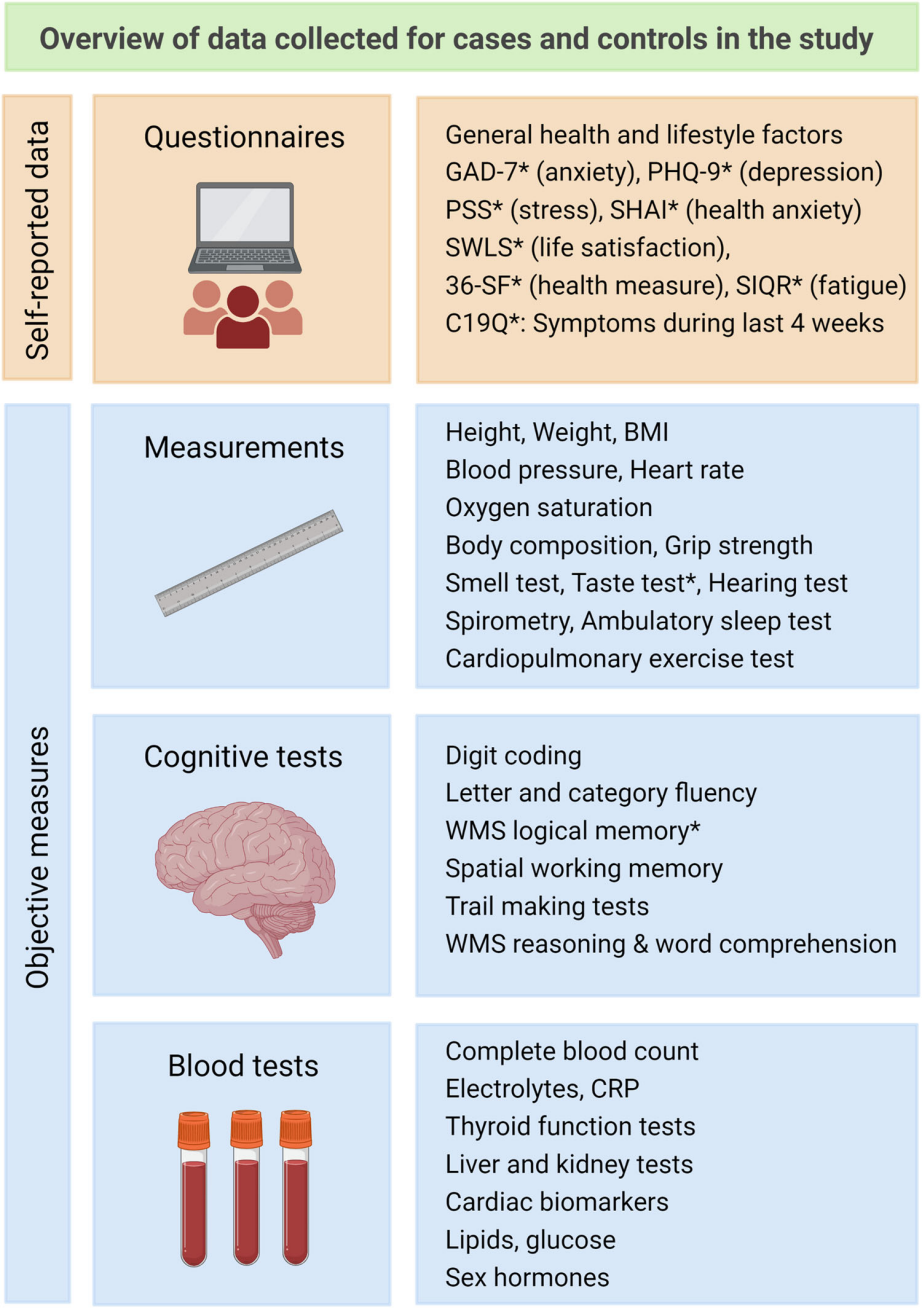
Overview of data collected in the study. Traits marked with * were added to the dHS Study in September 2020 for the dHS Covid Study and thus historical dHS control data were not available for those. Historical data was available from the iStopMM study for the GAD-7, PHQ-9, PSS, SWLS, and 36-SF questionnaires. A subset of the dHS historical controls answered the C19Q between September 2020 and November 2021 enriching the contemporary control data for C19Q. Images created with Biorender.com.

The study design is shown in Fig. 1, including the definition of contemporary and historical controls and the data used in assessment of longitudinal measures for a subset of cases and controls. Overview of data collection is presented in Fig. 2. The average age of the 1706 Covid cases was 46 years (range: 18–93) and 50% were women. The most common comorbidities were obesity (body mass index (BMI) ≥ 30; 33%), hypertension (19%), asthma (11%), and immunocompromised state (6%) (Table 1). Demographics and comorbidities were similar among those who participated and those who were invited but did not participate (Supplementary Data 1). We compared the cases to 14,398 controls, comprised of 13,779 historical and 619 contemporary study participants (Fig. 1). The average age of the controls was 56 years (range: 18–97), 57% were women, and hypertension, asthma, and immunocompromised state were more common among controls than cases (Table 1).

**Table 1:** Comparing sex, age, and comorbidities between cases, controls, and cases with long Covid.

	SARS-CoV-2 cases participating in the study	SARS-CoV-2 cases with long Covid	All controls	Contemporary controls	All C19Q controls	SARS-CoV-2 cases (1) vs all controls (0)		SARS-CoV-2 cases (1) vs contemporary controls (0)		SARS-CoV-2 cases (1) vs C19Q controls (0)		SARS-CoV-2 cases with long Covid (1) vs other cases (0)	
						Effect (95% CI)	*P* value	Effect (95% CI)	*P* value	Effect (95% CI)	*P* value	Effect (95% CI)	*P* value
*N* all	1706	490	14,398	619	1379								
Sex, *N* women (%)	856 (50%)	316 (64%)	8135 (57%)	309 (50%)	756 (55%)	0.78 (0.7–0.86)	8.0E-07	1.01 (0.84–1.21)	0.91	0.82 (0.71– 0.94)	0.0056	2.27 (1.82– 2.82)	8.2E-14
Age, mean (range)	45.6 (18–93)	46.7 (18–88)	55.5 (18–97)	47.6 (19–89)	47.6 (18–89)	−9.8 (−10.5 to −9.1)	8.7E-150	−1.9 (−3.3 to −0.6)	0.0059	−1.8 (−2.8 to −0.8)	0.00026	1.4 (−0.2–3.0)	0.084
Days since diagnosis, mean (range)	262 (146–557)	257 (147–541)										−6 (−14–1)	0.091
Obesity (BMI ≥ 30)	553 (33%)	217 (45%)	4911 (34%)	202 (33%)	464 (34%)	1.07 (0.96–1.19)	0.28	1.05 (0.86–1.28)	0.64	1.00 (0.86–1.17)	0.96	1.97 (1.57–2.47)	5.3E-09
Hypertension	298 (19%)	103 (22%)	5199 (36%)	133 (23%)	310 (23%)	0.70 (0.61–0.81)	1.5E-06	0.78 (0.60–1.02)	0.068	0.73 (0.60–0.90)	0.0031	1.37 (1.01–1.87)	0.05
Asthma	171 (11%)	75 (16%)	1993 (14%)	84 (14%)	192 (14%)	0.80 (0.68–0.95)	0.0093	0.72 (0.54–0.96)	0.023	0.75 (0.60–0.93)	0.008	1.95 (1.40–2.71)	8.2E-05
Immunocompromised state	59 (6%)	36 (12%)	1405 (10%)	29 (7%)	88 (8%)	0.75 (0.57–0.98)	0.037	0.79 (0.49–1.26)	0.32	0.76 (0.53–1.07)	0.11	3.58 (2.04–6.27)	8.3E-06
Cancer	75 (4%)	33 (7%)	1340 (9%)	26 (4%)	67 (5%)	0.82 (0.64–1.05)	0.068	1.12 (0.69–1.79)	0.29	0.89 (0.63–1.27)	0.16	1.8 (1.09–2.97)	0.021
Type 2 diabetes	77 (5%)	32 (7%)	1097 (8%)	32 (5%)	75 (5%)	0.81 (0.63–1.03)	0.078	0.90 (0.59–1.38)	0.62	0.88 (0.63–1.23)	0.43	1.64 (1.01–2.66)	0.044
Coronary artery disease	70 (4%)	30 (6%)	1463 (10%)	26 (4%)	62 (5%)	1.07 (0.96–1.19)	0.057	1.05 (0.86–1.28)	0.96	1.00 (0.86–1.17)	0.47	1.97 (1.57–2.47)	0.0033

Shown are associations of SARS-CoV-2 infection with sex, age, and comorbidities, using three different control groups; all controls, contemporary controls, and all controls answering the C19Q symptom questionnaire. Sex, age, time since diagnosis of SARS-CoV-2, and comorbidities were also compared between cases with long Covid and other SARS-CoV-2 cases. Association testing for sex was performed with logistic regression. For age and time since diagnosis the association testing was performed with linear regression. Association testing for comorbidities was performed with logistic regression, adjusting for age and sex. Effects for sex and comorbidities are given in odds ratios and betas are reported for age and time since diagnosis, and 95% confidence intervals (CI) are given for all effects. *P* values were obtained from a likelihood ratio test. Immunocompromised state was defined as immunocompromised from solid organ transplant, blood or bone marrow transplant, immune deficiencies, HIV, use of corticosteroids, or use of other immune weakening medicines (Supplementary Methods).

The severity of the acute SARS-CoV-2 infection ranged from no/mild illness (41%) to severe illness requiring hospitalization (5%) (Supplementary Table 1). Persons of older age were at higher risk of more severe acute infection as were those with obesity, asthma and several other medical conditions that have previously been associated with severity^36^ (Supplementary Data 1, Supplementary Methods). Levels of anti-N antibodies measured at the time of study visit correlated positively with the severity of the acute infection (Supplementary Figure 5). The duration from the diagnostic qPCR test to study visit ranged from 5–18 months (median = 253 days, range = 146–557 days) (Supplementary Figure 6).

### Self-reported data

Asked specifically about recovery from the acute illness, 23% of cases reported not having recovered and 5% reported still having severe symptoms five to six months after infection. These fractions decreased to 15% and 1%, respectively, 13 months after infection. One in four of all cases had sought medical attention for residual symptoms.

**Fig. 3:**
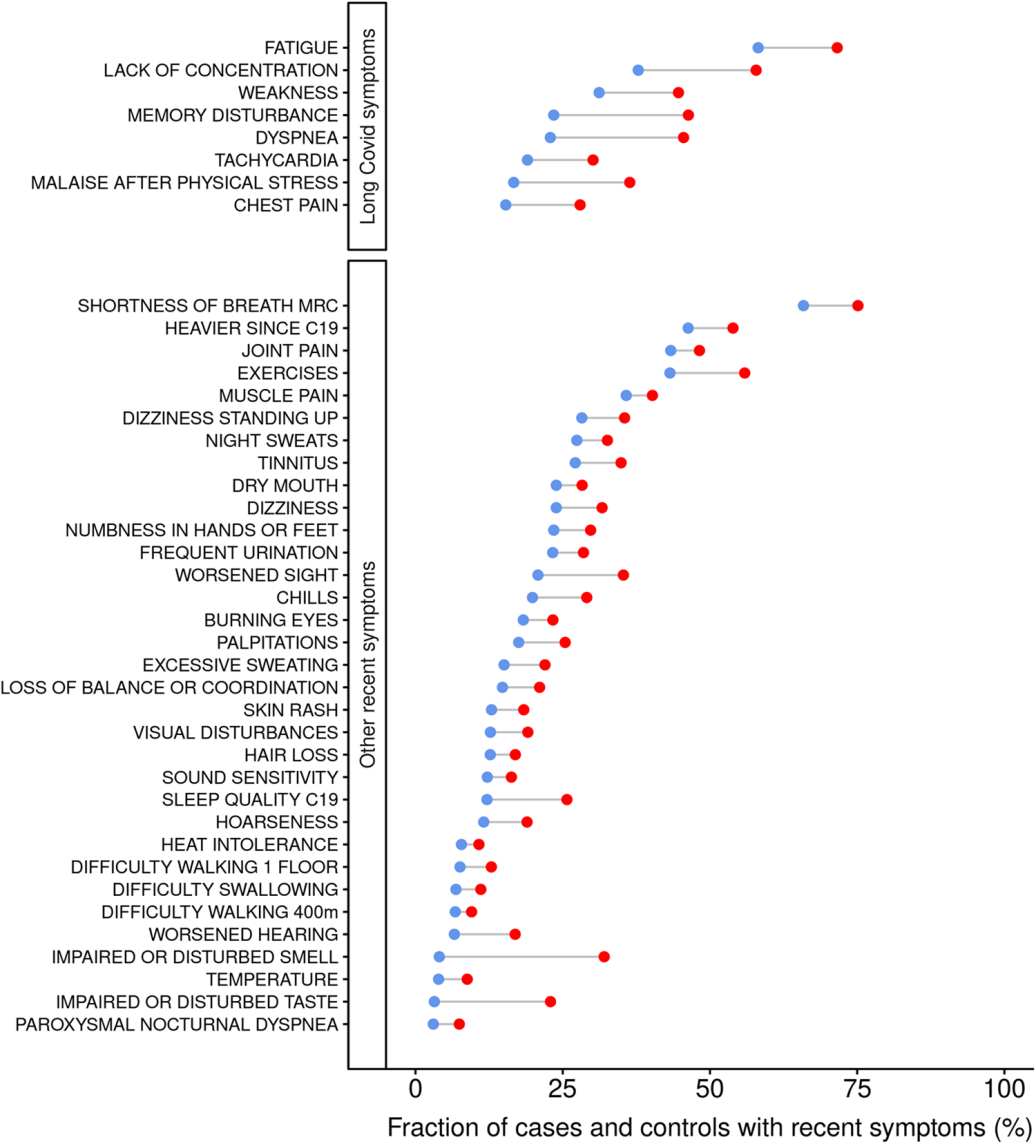
The fraction of cases and controls reporting the 41 recent symptoms associated with prior SARS-CoV-2 infection. All 1706 cases and 1379 C19Q controls answered the C19Q questionnaire between September 2020 and November 2021. Participants were asked about the frequency and/or severity of symptoms during the 4 weeks prior to answering (details about prevalence definitions are found in Supplementary Data 2). Red dots denote cases and blue dots denote controls and results are sorted by fraction of controls reporting symptoms, in descending order within panels. The symptoms included in the long Covid definition are grouped together on the *y* axis.

All 1706 cases and 619 contemporary controls answered questions in the C19Q questionnaire about frequency and/or severity of specific symptoms during the four weeks prior to study visit. In addition, an online version of the C19Q was sent to a subset of the historical controls, 2000 persons, age and sex-matched to the cases, and 760 responded between September 2020 and November 2021, resulting in a control group of 1379 persons for the C19Q symptom data. Cases reported more symptoms than controls, with 41 out of 88 symptoms (47%) associating with prior SARS-CoV-2 infection (*P* < 5 × 10^−4^, Fig. 3, Supplementary Data 2). These are symptoms experienced by cases 5–18 months after the acute infection.

The symptoms associating most significantly with prior SARS-CoV-2 infection were disturbed smell and taste, memory disturbance, and dyspnea. Cases reported disturbed smell and taste 12 and 10 times more frequently than controls, respectively, and memory disturbance and dyspnea three times more commonly (Fig. 3, Supplementary Data 2). It is worth noting that disturbed smell and taste were uncommon among controls, with a prevalence of 4% and 3%, respectively, while memory disturbance and dyspnea were common, with a prevalence of 24% and 23%, respectively (Fig. 3, Supplementary Data 2). The prevalence of seven symptoms changed with time from the acute infection (*P* < 0.05); smell and taste improving but others including malaise after physical exertion worsening (Supplementary Data 2).

We noted a large cluster of correlated symptoms including dyspnea, fatigue, weakness, malaise after physical exertion, memory disturbance, and lack of concentration (Pearson correlation coefficient (*r*): 0.37–0.68, Supplementary Figure 7). Impaired or disturbed smell and taste correlated highly with each other (*r* = 0.78) and considerably less with other symptoms (*r* < 0.26), but highest with memory disturbance (*r* = 0.24 and 0.26, respectively).

Our evaluation of mental health and quality of life with validated questionnaires (Fig. 2) showed less symptoms of stress (PSS^20^) among cases than controls (*P* < 5 × 10^−4^). Longitudinal measures demonstrated less symptoms of stress among both cases and controls during the pandemic than before it, but the reduction in symptoms of stress was larger in cases than controls (Table 2, Supplementary Data 3). We did not find differences between cases and controls for symptoms of anxiety (GAD-7^18^), health anxiety (SHAI^21^), depression (PHQ-7^19^), fatigue (SIQR^22^), satisfaction of life (SWLS^23^), or health-related quality of life (36-SF^24^) in these data (Supplementary Data 3).

**Table 2:** Test measures that associate with prior SARS-CoV-2 infection.

		(1) SARS-CoV-2 cases vs all controls			(2a) SARS-CoV-2 cases vs contemporary controls			(2b) The effect of SARS-CoV-2 status on the difference between pandemic and pre-pandemic measurements		
Test, trait (unit)		Cases/ctrls	Effect (95% CI)	*P* value	Cases/ctrls	Effect (95% CI)	*P* value	Cases/ctrls	Effect (95% CI)	*P* value
Perceived Stress Scale (score)	Q	901/34391	−1.97 (−2.37 to −1.57)	2.7E-22	1487/616	−0.37 (−1.00–0.26)	0.25	264/19859	−1.63 (−2.29 to −0.97)*	1.2E-6
Smell, selective anosmia	B	1668/14090	3.72 (3.46–3.99)	9.5e-19	1668/617	4.29 (3.56–5.03)*	2.7E-6	122/294	0.06 (0.02–0.11)*	0.0099
Smell, hyposmia	B	1668/14090	1.88 (1.73–2.04)	1.0E-13	1668/617	2.61 (2.24–2.97)*	2.0E-8	122/294	0.12 (0.04–0.20)*	0.0036
Taste, selective ageusia	B	1669/615	2.44 (1.93–2.95)	1.8E-4	1669/615	2.44 (1.93–2.95)	1.8E-4	NA^$^	NA^$^	NA^$^
Grip strength (kg)	Q	1670/8154	−0.71 (−1.10 to −0.31)	4.9E-4	1670/614	−0.66 (−1.41–0.03)*	0.062	59/139	0.45 (−1.50–2.40)	0.65
Delayed memory recall^#^ (SD)	Q	1602/602	−0.24 (−0.34 to −0.14)	3.3E-6	1602/602	−0.24 (−0.34 to −0.14)	3.3E-6	NA^$^	NA^$^	NA^$^
Immediate memory recall^#^ (SD)	Q	1602/602	−0.18 (−0.28 to −0.08)	3.2E-4	1602/602	−0.18 (−0.28 to −0.08)	3.2E-4	NA^$^	NA^$^	NA^$^

The association testing was performed using logistic regression for binary traits (B) and linear regression for quantitative traits (Q), adjusting for sex and age, and also for education when testing for association with memory recall. Effects for binary traits are given in odds ratios (OR) in columns 1 and 2a but as betas in column 2b. *P* values are obtained from a likelihood ratio test. Shown are all associations that are significant when comparing cases with all available controls, accounting for multiple testing (1). Consistent association results with (1) when comparing cases with contemporary controls (same direction and non-heterogeneity in the effect estimates) are marked with * in (2a) and significant effects (same direction) of SARS-CoV-2 status on the difference between longitudinal measures for cases and controls (2b), are marked with * in (2b).

*CI* 95% confidence interval. *CPET* Cardiopulmonary exercise test. ^#^Wechsler Memory Scale Logical Memory recall. ^$^Taste and delayed memory recall were not tested prior to the pandemic. *SD* standard deviations.

### Physiological tests

The physiological test traits that associated with prior SARS-CoV-2 infection, accounting for multiple testing (*P* < 6 × 10^−4^), were smell, taste, and grip strength (Table 2, Supplementary Data 4).

Several test measures reflecting disturbed smell and taste were more common among cases than controls, including hyposmia (based on 10th percentile cutoff on intensity ratings for six odors), selective anosmia (loss of smell for one or more odors), and selective ageusia (loss of taste for one or more tastants, Supplementary Methods, Table 2). Cases performed worse in odor identification and reported lower pleasantness ratings for most odors than controls, with the intensity rating of lemon odor being most different (Supplementary Data 4). Comparison of longitudinal smell test results for 122 cases showed worse results after infection for many smell measures, confirming the effect of the infection (Table 2, Supplementary Table 2). Several measures of smell improved with time from the acute infection such as hyposmia which was equally common among cases and controls nine months after infection (Supplementary Data 4). We did not see improvements of selective anosmia or selective ageusia with time among cases.

Cases had less grip strength than all controls (−0.71 kg, 95% CI: −1.10 to −0.31, Table 2) with consistent results when cases were compared to the smaller contemporary control group.

No other physiological test measures associated with prior SARS-CoV-2 infection accounting for age, sex, and multiple testing, including BMI, oxygen saturation, blood pressure, heart rate, heart rate variability, orthostatic hypotension, postural orthostatic tachycardia, exercise capacity, hearing, and spirometry (Supplementary Data 4, Fig. 2, Supplementary Methods).

### Cognitive tests

We compared 12 measures from six cognitive tests (Fig. 2) between cases and controls, using contemporary and historical control data for all tests except the Wechsler Memory Scale (WMS) Logical Memory tests for which only contemporary control data existed (Supplementary Data 5). Cases performed worse than controls on the WMS Logical Memory tests of both delayed memory recall (−0.24 standard deviations (SD), 95% CI: −0.34 to −0.14) and immediate memory recall (−0.18 SD, 95% CI: −0.28 to −0.08) accounting for multiple testing (*P* < 6 × 10^−4^, Table 2, Supplementary Data 5).

We calculated the prevalence of memory impairment, defined as a z-score of less than or equal to −1.5, among cases and controls, adjusting for age, gender, and educational level^37^. The prevalence of impairment in delayed memory recall was 12.5% for cases and 7.4% for controls (OR = 1.78, 95% CI: 1.43 to 2.15, *P* = 9.9 × 10^−4^) and the prevalence of impairment in immediate memory recall was 9.8% for cases and 8.7% for controls, respectively (OR = 1.13, 95% CI: 0.78–1.48, *P* = 0.48). Restricting the analysis to cases with symptoms of memory disturbance at least five times per week yielded a prevalence of impairment of 15.2% for delayed recall and 14.0% for immediate recall.

### Blood test results

We compared multiple blood tests between cases and contemporary controls and none associated with prior infection, including C-reactive protein, white blood cell count, and conventional cardiac, kidney, liver, and thyroid function tests, accounting for multiple testing (*P* < 8 × 10^−4^, Supplementary Data 6).

### Association of symptoms and test measures with severity of the acute infection

We tested for association of measures with severity of the acute infection and required *P* < 0.05 to establish association for measures that associated with prior infection in our study. If association with prior infection had not been demonstrated, we required the same multiple testing thresholds as listed above to establish association with severity of the acute infection.

All but one SARS-CoV-2 associating symptom (skin rash) correlated also with severity of the acute infection (*P* < 0.05) with malaise after physical exertion, dyspnea, and correlating symptoms associating most significantly (Supplementary Data 2). Impaired smell and taste associated considerably less significantly with severity of the acute infection. Similarly, more stress (PSS, *P* < 0.05), anxiety (GAD-7), health anxiety (SHAI), depression (PHQ-7), and fatigue (SIQR), as well as less satisfaction of life (SWLS) and poorer health-related quality of life (36-SF) associated with more severe acute illness (*P* < 5 × 10^−4^, Supplementary Data 3).

The three physiological test measures that associated with prior infection, impaired smell, impaired taste, and lower grip strength, associated also with more severe acute illness (*P* < 0.05) (Supplementary Data 4). Other physiological measures associating with severity were higher fat mass index (FMI), BMI and lean mass index, and lower exercise capacity measured by lower oxygen consumption (VO2) at maximal exertion in a cardiopulmonary exercise test (VO2 max) (*P* < 6 × 10^−4^). The WMS Logical Memory tests of immediate and delayed recall did not associate with severity (*P* > 0.05) (Supplementary Data 5).

Lower HDL-cholesterol and Apolipoprotein A, and higher triglyceride levels associated with more severe acute infection (*P* < 8 × 10^−4^) (Supplementary Data 6).

### Long Covid symptom cluster

29% of cases (28% of non-hospitalized cases) and 16% of controls met our long Covid symptom criteria. Cases were 2.1 (95% CI, 1.6 to 2.7) times more likely to fulfill the criteria than controls. Adjustment for comorbidities increased the odds ratio to 2.9 (95% CI, 2.1 to 4.0). Of cases fulfilling the criteria, 54% had to reduce their regular hours for work, school, household, or other, due to persistent symptoms or complications from their SARS-CoV-2 infection, compared to 23% of cases overall. This translates to a long Covid prevalence of 7% according to the WHO case definition^35^. Women were more likely to satisfy the long Covid criteria and other associating factors were more severe acute infection and several medical conditions including heart failure, immunocompromised state, and coronary artery disease, but not time from the infection (Table 1 and Supplementary Data 1).

We explored the association of long Covid with physiological, cognitive, and blood traits in cases and controls separately (Supplementary Data 7). We found that among both cases and controls the criteria associated most significantly with lower exercise capacity (V02 max), higher BMI and FMI, and with impaired smell and taste among cases.

### Transparency declaration

The lead authors, Kari Stefansson and Hilma Holm, affirm that this manuscript is an honest, accurate, and transparent account of the study being reported; that no important aspects of the study have been omitted; and that any discrepancies from the study as planned (and, if relevant, registered) have been explained.

## Discussion

We performed a detailed assessment of 1706 persons with a confirmed SARS-CoV-2 infection 5–18 months prior and compared to 14,398 controls. A wide variety of symptoms associated with prior infection and for objective measures associations were observed for smell, taste, grip strength, and memory recall.

Our case population represents the severity spectrum of the acute SARS-CoV-2 infection, ranging from no or very mild symptoms to severe illness with 5% hospitalized in the acute phase^2,34,38^. It follows that our study population consists mainly of persons who did not require hospitalization during acute illness. The comorbidities that predisposed to severe acute infection in our sample were the same as others have reported^36^, suggesting that our sample is representative of those infected with SARS-CoV-2. Based on self-report, 15% had not recovered and 1% still suffered severe symptoms 13 months after the infection.

Deficits in smell and taste are common symptoms of the acute SARS-CoV-2 infection^39^ and reports suggest full recovery in most at six months^40,41^. We found both subjective and objective measures of smell and taste impairment to be more common among cases than controls in our study, with the slow improvement of symptoms with time. Some test results of smell and taste improved with time, with hyposmia normalizing at nine to 10 months after infection, but selective anosmia and selective ageusia did not improve.

Sensorineural hearing loss is a recognized complication of viral infections and there are multiple reports of hearing loss in persons with a history of SARS-CoV-2, with most studies based on self-reported questionnaires or medical reports without conclusive hearing tests^42^. Here, cases noted worsening of hearing from before the pandemic four times more often than controls, with half of them linking the noted change to the infection. However, hearing test results did not associate with a history of SARS-CoV-2. Thus, we do not have objective evidence of SARS-CoV-2 causing hearing loss among our mostly non-hospitalized patients.

We observed lower grip strength in persons with prior SARS-CoV-2 infection, possibly due to deconditioning. Grip strength, a measure of muscle strength, is a strong predictor of cardiovascular disease and mortality^43^, and indeed incident cardiovascular outcomes appear to be more common in survivors of acute Covid-19 than controls^44^. It should be noted that although significant, the difference in grip strength between cases and controls was small. We did not observe association between prior infection and exercise capacity as measured with a cardiopulmonary exercise test.

It has been suggested that chronic myocardial inflammation is a common complication of SARS-CoV-2 infection, irrespective of both pre-existing conditions and severity of the acute infection^45–47^. One case-control study, assessing 443 individuals after SARS-CoV-2 infection, reported a small reduction in left ventricular ejection fraction and higher concentration of hs-TNT and NT-proBNP after infection compared to controls^47^. Complicating the interpretation of that study is the lack of contemporary controls, as the control data were derived from persons assessed prior to the pandemic, precluding exploration of time trends in measures as well as any effect of the pandemic itself. The lack of association of the cardiac biomarkers hs-TNT and NT-proBNP with history of SARS-CoV-2 infection in our study of 1706 cases, argues against persistent myocardial involvement as a common complication of milder infections. Similarly, we found no evidence of persistent systemic inflammation, hematologic abnormalities, kidney, or liver dysfunction using conventional blood biomarkers.

There have been several reports of dysautonomia after SARS-CoV-2 infection, in particular orthostatic intolerance. We assessed several measures of dysautonomia in our study, including heart rate variability, orthostatic hypotension, and postural orthostatic tachycardia, and found no association with prior infection. Marques et al compared 155 select individuals with long Covid to 94 controls and found they had reduced heart rate variability^48^. In our study, we measured heart rate variability during sleep and found no association with either history of infection (402 individuals) or long Covid (706 individuals). The discrepancy between the two studies could be explained by different selection of cases or different heart rate variability measurement or both.

We performed extensive cognitive testing and observed that poorer delayed and immediate memory recall associated with prior SARS-CoV-2 infection, but the effects were small. We calculated the prevalence of impairment in delayed memory recall as 12.5% for cases and 7.4% for controls providing an objective measure of the memory disturbance commonly reported after infection. The prevalence of memory recall impairment in our study is comparable to the 12% reported by Becker et al^37^ for non-hospitalized persons. However, we did not find significant differences between cases and controls in other cognitive tests, unlike Becker and colleagues who reported high prevalence of cognitive impairment of many domains in their smaller study of 740 persons evaluated after Covid-19 in a clinical setting.

The protracted symptoms of fatigue and neurocognitive disturbance after SARS-CoV-2 infection are reminiscent of other post-infective fatigue syndromes and myalgic encephalomyelitis/chronic fatigue syndrome (ME/CFS)^49–51^. The subjective cognitive impairments that are some of the more debilitating symptoms of ME/CFS^52^ have been captured by objective measures^53^ but discrepancies between symptoms and test results are common, possibly due to inability of cognitive tests to capture mild impairment and deficits affecting real-life tasks^54^. The WMS Logical Memory test may be a relatively sensitive measure of cognitive deficits following SARS-CoV-2 infection, as it has previously been used to detect subtle changes in memory^55^. Furthermore, the pathogenesis of these symptoms is unclear. A study leveraging longitudinal brain imaging data from the UK Biobank reported changes in brain structure associated with prior infection with SARS-CoV-2 but changes in areas related to memory did not associate with cognitive tests results^56^. It is also notable that while symptoms of neurocognitive disturbance associated with severity of the acute infection in our study, measured deficits in memory recall do not.

Higher BMI, higher triglycerides, and lower HDL-C levels associated with the severity of the acute infection but not with prior SARS-CoV-2 infection per se. These are all risk factors for more severe acute infection^57^, and thus the observed association likely reflects the predisposition, rather than being a consequence of more severe infection. We replicate the association of more anxiety and depression with more severe infection^47^ but given the lack of association between these phenotypes and prior infection, the causal nature of this relationship is not clear.

The high prevalence of symptoms among cases compared to controls contrasts notably with the small difference observed in test measures between the two groups, as well as with discrepancies between some symptoms and related test measures. For example, tachycardia was a prominent symptom after infection but there was no significant difference in measured heart rate between the two groups. Cases reported gaining weight since before the pandemic more often than controls, but there was no difference in BMI between cases and controls, or in longitudinal measures for cases. Similar observations for hearing are described above. Measured memory impairment was 1.78-fold more common in cases than controls while self-reported memory disturbance was described 3.5-fold more commonly by the cases. These observations support an element of response bias^58^ in self-reported symptoms following SARS-CoV-2 infection and a more complicated biological or biopsychosocial contribution to the persistent symptoms^59^ that are not well captured by conventional tests. These are important considerations for both research and clinical assessment of post-Covid conditions. Conventional clinical assessment would thus not be expected to be particularly informative in relating reported symptoms to a past SARS-CoV-2 infection.

There are extensive research efforts ongoing worldwide aiming to increase our understanding of the pathophysiology of illness following infection with the SARS-CoV-2 virus and several mechanisms have been identified as potential mediators or culprits, including immune dysregulation, viral persistence, and endothelial dysfunction. Hopefully, this research will lead to improved diagnostics and effective treatments of this heterogenous condition^60^.

Our attempt to estimate the prevalence of long Covid, using data for both cases and controls, highlights not only how common the symptoms of long Covid are in the general population but also the importance of control data for comparison. The excess of cases meeting our criteria for long Covid was 13% with half of those reporting impact on everyday function, translating to a long Covid prevalence of 7% at a median of 8 months after infection. These estimates do not account for potential biases in self-reported symptoms. The lack of a universal and uniform definition of long Covid is problematic for both research and health care and complicates comparison between studies and populations. Indeed, prevalence estimates for long Covid in non-hospitalized cohorts have a wide range, up to at least 52% when the presence of one persisting symptom is sufficient for the diagnosis^13^. The approach by the Coronavirus (COVID-19) Infection Survey in the UK was similar to ours and reported a 3% prevalence of continuous symptoms after infection compared to 0.5% of controls^11^.

This study has limitations. First, although more than half of Icelanders diagnosed with SARS-CoV-2 infection before October 2020 participated in the study, participation bias cannot be excluded and it is plausible that cases with more pronounced symptoms were more likely to participate, although demographics and comorbidities were similar among those who participated and those who did not. Second, while the study represents adults of all ages, it does not include children. Third, while the availability of measures before and after the infection with similar longitudinal measures for controls is a particular strength of the study, this sample set was relatively small. Fourth, this is a study of Icelanders, a North European ethnic group, in Iceland, a wealthy country with universal access to health care. The study results may not apply to persons of other origins and/or in different circumstances. Fifth, the study includes cases infected with SARS-CoV-2 before vaccinations started. Last, with regard to the objective traits assessed in our study, we can only assess what we measure.

We believe that the inclusion of both historical and contemporary controls is a major strength of our study, allowing for consideration of possible time effects, i.e., general consequences of the pandemic itself (social isolation, reduced mobility) in addition to direct effects of the SARS-CoV-2 infection (viral invasion, resulting illness).

In conclusion, in our comprehensive case-control study of mostly non-hospitalized Icelanders, multiple and diverse symptoms were more common among cases than controls 5–18 months after SARS-CoV-2 infection. However, objective differences between cases and controls in the parameters we measured were few. Cases performed worse than controls in tests of smell and taste with improvement in some of these measures over time. Cases also performed worse in tests of grip strength and immediate and delayed memory recall, but here differences between cases and controls were small. We show that many symptoms associated with prior SARS-CoV-2 infection are common in the general population and, accounting for that, estimate the prevalence of long Covid to be ~7%. Discrepancies between symptoms and objective measures suggest an element of response bias in self-reported symptoms and a more complicated contribution to symptoms related to prior infection than is captured by conventional tests.

## Supplementary information

Description of Additional Supplementary Files

Supplementary Information

Supplementary Data 1

Supplementary Data 2

Supplementary Data 3

Supplementary Data 4

Supplementary Data 5

Supplementary Data 6

Supplementary Data 7

Reporting Summary

## Data Availability

In order to comply with the approval by the Icelandic National Bioethics Committee for this study and the provisions for the processing of personal data in deCODE genetics research issued by the Icelandic Data Protection Authority, individual participant data from the deCODE Health Study will not be made available to others. The study protocol and statistical analysis plan are described in the main manuscript and Supplementary Information and more detailed information is available upon reasonable request. Detailed data on the demographics and comorbidities of study participants are shown in Supplementary Data 1. Data underlying Figs. 3 are found in Supplementary Data 2. Detailed results of the association between prior SARS-CoV-2 infection and various metrics are found in Supplementary Data: association with metrics of mental health and quality of life are presented in Supplementary Data 3, with physiologic test traits in Supplementary Data 4–6. The association of long Covid with physiological, cognitive, and blood traits is presented in Supplementary Data 7.
