# Supplementary material for: Physical and cognitive impact following SARS-CoV-2 infection in a large population-based case-control study
Source: Commun Med (Lond). 2023 Jul 6;3:94. doi: 10.1038/s43856-023-00326-5 (PMC10325957; doi:10.1038/s43856-023-00326-5)
Supplement: Supplementary file 1 — Description of Additional Supplementary Files [file 43856_2023_326_MOESM1_ESM.pdf]

Description of Additional Supplementary Files

File Name: Supplementary Data 1

Description: Demographics and comorbidities. Shown are the associations of prior SARS-CoV-2 infection with sex, age and comorbidities using three different control groups; all controls, contemporary controls only and C19Q controls. Sex, age and comorbidities are also compared between cases that participated in our study vs those that were invited but did not participate (this comparison is performed for those that were infected during the first wave of the pandemic in Iceland, in March or April, 2020, since they were all invited to the study; 68% of the cases in the study were infected during the first wave). The association of the long Covid symptom cluster is shown for sex, age, time since diagnosis of SARS-CoV-2 infection and comorbidities, among cases and controls separately. Association testing for sex was performed with logistic regression. For age and time since diagnosis the association testing was performed with linear regression. Association testing for comorbidities was performed with logistic regression, adjusting for age and sex. The last column shows the association of the severity scale (severity of the acute phase of the infection) with sex, age, time since diagnosis of SARS-CoV-2 infection and comorbidities. The association between comorbidities and the severity scale was performed with linear regression, regressing the severity scale against comorbidities, adjusting for age and sex. Effects from logistic regression are given in odds ratios (OR) and 95% confidence intervals (CI) are given for all effects. P-values were obtained from a likelihood ratio test, and associations marked with * have P-value < 0.05 and those marked with ** have P-value < 0.001. Immunocompromised state was defined as immunocompromised from solid organ transplant, blood or bone marrow transplant, immune deficiencies, HIV, use of corticosteroids, or use of other immune weakening medicines (Supplementary Methods). Severity scale ranges from 0 (least severe acute infection) to 8 (most severe acute infection). In the severity categories we have grouped the severity scales such that two groups from the severity scale are in each category and 1 equals least severe acute infection and 4 most severe acute infection.

File Name: Supplementary Data 2

Description: Symptoms assessed with the C19Q questionnaire.
The table shows the association of SARS-CoV-2 status, the severity scale and severity categories with recent symptoms, using logistic regression adjusting for age and sex (and comorbidities, when specified). For logistic regression analysis, the symptoms (symptoms during the four weeks prior to study participation) are coded as 1 or 0 as is defined in the last column. The association between time since diagnosis and symptoms, was performed using a linear regression adjusting for age and sex (and comorbidities, when specified). P-values are obtained with a likelihood ratio test. To establish association of symptoms and test measures with prior SARS-CoV-2 infection, we compared cases with all available controls and accounted for multiple testing, arriving at $P < 5 \times 10^{-4}$ for health and symptom questionnaire data. Severity scale ranges from 0 (least severe acute infection) to 8 (most

severe acute infection). In the severity categories we have grouped the severity scales such that two groups from the severity scale are in each category and 1 equals least severe acute infection and 4 most severe acute infection.

File Name: Supplementary Data 3

Description: Validated mental health, fatigue and quality of life questionnaire scales. The table shows the association of SARS-CoV-2 status, the severity scale, severity categories and time since diagnosis with scores from questionnaires, using linear regression adjusting for age and sex (and comorbidities when specified), as well as comparison of scores for individuals that had answers from both before and during the pandemic (obtained with data from the iStopMM study). The SIQR score was obtained from the C19Q questionnaire, while other scores were obtained from the online questionnaire. P-values are obtained with a likelihood ratio test. To establish association of symptoms and test measures with prior SARS-CoV-2 infection, we compared cases with all available controls and accounted for multiple testing, arriving at $P < 5 \times 10^{-4}$ for health and symptom questionnaire data. To take into account possible confounding of time in the effect estimates for SARS-CoV-2 status, e.g., via simultaneous pandemic effects, we required consistent results (same direction and non-heterogeneity in effect estimates) when the analysis was restricted to contemporary controls or same direction in the effect estimates in the comparison of longitudinal measures between cases and controls (Figure 1). Severity scale ranges from 0 (least severe acute infection) to 8 (most severe acute infection). In the severity categories we have grouped the severity scales such that two groups from the severity scale are in each category and 1 equals least severe acute infection and 4 most severe acute infection.

File Name: Supplementary Data 4

Description: Physiological test measures. The associations of SARS-CoV-2 status, the severity scale, severity categories and time since diagnosis with physiological measures. The association of SARS-CoV-2 status was performed separately using all available controls and only using contemporary controls. Furthermore, historic controls were compared to contemporary controls. For binary traits, the effect is an OR from a logistic regression, adjusting for age and sex (and comorbidities when specified). For quantitative traits, the effect is from a linear regression, adjusting for age and sex (and comorbidities when specified). P-values are obtained with a likelihood ratio test. To establish association of symptoms and test measures with prior SARS-CoV-2 infection, we compared cases with all available controls and accounted for multiple testing, arriving at $P < 6 \times 10^{-4}$ for physiological test results. To take into account possible confounding of time in the effect estimates for SARS-CoV-2 status, e.g., via simultaneous pandemic effects, we required consistent results (same direction and non-heterogeneity in effect estimates) when the analysis was restricted to contemporary controls or same direction in the effect estimates in the comparison of longitudinal measures between cases and controls (Figure 1). Severity scale ranges from 0 (least severe acute infection) to 8 (most severe acute infection). In the severity categories

we have grouped the severity scales such that two groups from the severity scale are in each category and 1 equals least severe acute infection and 4 most severe acute infection.

File Name: Supplementary Data 5

Description: Cognitive test measures. The associations of SARS-CoV-2 status, the severity scale, severity categories and time since diagnosis with cognitive measures. The association of SARS-CoV-2 status was performed separately using all available controls and only using contemporary controls. Furthermore, historic controls were compared to contemporary controls. Effects are from a linear regression and in SD units, adjusting for age, sex and level of education (and comorbidities when specified). P-values are obtained with a likelihood ratio test. To establish association of symptoms and test measures with prior SARS-CoV-2 infection, we compared cases with all available controls and accounted for multiple testing, arriving at $P < 6 \times 10^{-4}$ for cognitive test results. To take into account possible confounding of time in the effect estimates for SARS-CoV-2 status, e.g., via simultaneous pandemic effects, we required consistent results (same direction and non-heterogeneity in effect estimates) when the analysis was restricted to contemporary controls or same direction in the effect estimates in the comparison of longitudinal measures between cases and controls (Figure 1). Severity scale ranges from 0 (least severe acute infection) to 8 (most severe acute infection). In the severity categories we have grouped the severity scales such that two groups from the severity scale are in each category and 1 equals least severe acute infection and 4 most severe acute infection.

File Name: Supplementary Data 6

Description: Blood tests. The associations of SARS-CoV-2 status, the severity scale, severity categories and time since diagnosis with blood tests. Effects are from a generalized additive models (gam() in R mcgv package), adjusting for age, sex, a smoothing function of month of measure indicator (and comorbidities when specified). P-values are obtained with a likelihood ratio test. To establish association of symptoms and test measures with prior SARS-CoV-2 infection, we compared cases with all available controls and accounted for multiple testing, arriving at $P < 8 \times 10^{-4}$ for blood tests. Severity scale ranges from 0 (least severe acute infection) to 8 (most severe acute infection). In the severity categories we have grouped the severity scales such that two groups from the severity scale are in each category and 1 equals least severe acute infection and 4 most severe acute infection.

File Name: Supplementary Data 7

Description: Long Covid and physiological, cognitive, and blood tests. The effect of the long Covid symptom cluster on physiological and cognitive traits. For binary traits, the effect is an OR from a logistic regression, regressing the trait against the symptom cluster status (1 for individuals that have any of the symptoms, 0 otherwise). For quantitative traits, the effect is from a linear regression, regressing the trait against the symptom cluster status. Results are shown for associations among cases and controls separately. Results are shown when

adjusting for age and sex only and when adjusting for age, sex and comorbidities; obesity, asthma, hypertension, coronary artery disease, type 2 diabetes, and cancer (except we did not adjust for obesity when analyzing BMI and obesity as a trait). P-values are obtained with a likelihood ratio test.
