# Supplementary material for: Physical and cognitive impact following SARS-CoV-2 infection in a large population-based case-control study
Source: Commun Med (Lond). 2023 Jul 6;3:94. doi: 10.1038/s43856-023-00326-5 (PMC10325957; doi:10.1038/s43856-023-00326-5)
Supplement: Supplementary file 2 — Supplementary Information [file 43856_2023_326_MOESM2_ESM.pdf]

Supplementary Information

This information has been provided by the authors to give readers additional information about their work.

Supplement to:

Holm H, Ivarsdottir EV, Olafsdottir Th, et al.

Physical and cognitive impact following SARS-CoV-2 infection in a large population-based case-control study

Symptoms, physical measures and cognitive tests after SARS-CoV-2 infection in a large population-based case-control study

Hilma Holm[1]*, Erna V. Ivarsdottir[1]*, Thorhildur Olafsdottir[1]*, Rosa Thorolfsdottir[1], Elias Eythorsson[2], Kristjan Norland[1], Rosa Gisladottir[1,3], Gudrun Jonsdottir[1], Unnur Unnsteinsdottir[1], Kristin E. Sveinsdottir[1], Benedikt A. Jonsson[1], Margret Andresdottir[1], David O. Arnar[1,2,4], Asgeir O. Arnthorsson[1], Kolbrun Birgisdottir[1], Kristbjorg Bjarnadottir[1], Solveig Bjarnadottir[2,4], Gyda Bjornsdottir[1], Gudmundur Einarsson[1], Berglind Eiriksdottir[1], Elisabet Eir Gardarsdottir[1], Thorarinn Gislason[2,4], Magnus Gottfredsson[2,4], Steinunn Gudmundsdottir[1], Julius Gudmundsson[1], Kristbjorg Gunnarsdottir[1], Anna Helgadottir[1], Dadi Helgason[2], Ingibjorg Hinriksdottir[5], Ragnar F. Ingvarsson[2] , Sigga S. Jonasdottir[1], Ingileif Jonsdottir[1], Tekla H. Karlsdottir[1], Anna M. Kristinsdottir[1], Sigurdur Yngvi Kristinsson[2,4], Steinunn Kristjansdottir[1], Thorvardur J. Love[2,4], Dora Ludviksdottir[2,4], Gisli Masson[1], Gudmundur Norddahl[1], Thorunn Olafsdottir[1], Isleifur Olafsson[6], Thorunn Rafnar[1], Hrafnhildur L. Runolfsdottir[2], Jona Saemundsdottir[1], Svanur Sigurbjornsson[1], Kristin Sigurdardottir[1], Engilbert Sigurdsson[4,7], Martin I. Sigurdsson[4,8], Emil L. Sigurdsson[9,10], Valgerdur Steinthorsdottir[1], Gardar Sveinbjornsson[1], Emil A. Thorarensen[1], Bjarni Thorbjornsson[1], Brynja Thorsteinsdottir[1], Vinicius Tragante[1], Magnus O. Ulfarsson[1,11], Hreinn Stefansson[1], Thorsteinn Gislason[1], Mar Kristjansson[2], Runolfur Palsson[2,4], Patrick Sulem[1], Unnur Thorsteinsdottir[1,4], Gudmundur Thorgeirsson[1,4], Daniel F. Gudbjartsson[1,12], Kari Stefansson[1,4].

1. deCODE genetics/Amgen Inc., Reykjavik, Iceland
2. Internal Medicine and Emergency Services, Landspitali – The National University Hospital of Iceland, Reykjavik, Iceland
3. School of Humanities, University of Iceland, Reykjavik, Iceland
4. Faculty of Medicine, School of Health Sciences, University of Iceland, Reykjavik, Iceland.
5. National Institute of Hearing and Speech in Iceland, Reykjavik, Iceland
6. Clinical Laboratory Services, Diagnostics and Blood Bank, Landspitali – The National University Hospital of Iceland, Reykjavik, Iceland
7. Mental Health Services, Landspitali – The National University Hospital of Iceland, Reykjavik, Iceland
8. Perioperative Services, Landspitali – The National University Hospital of Iceland, Reykjavik, Iceland
9. Department of Family Medicine, University of Iceland, Reykjavik, Iceland
10. Development Centre for Primary Health Care in Iceland, Reykjavik, Iceland
11. Faculty of Electrical and Computer Engineering, University of Iceland, Reykjavik, Iceland
12. School of Engineering and Natural Sciences, University of Iceland, Reykjavik, Iceland

* These authors contributed equally.

Table of Contents

Supplementary Methods 5
Overview of the deCODE Health Study and changes to the study to address consequences of SARS-CoV-2 infection 5
The dHS online questionnaire on health and lifestyle 5
General Anxiety Disorder-7 6
Patient Health Questionnaire-9 6
Perceived Stress Scale 6
Short Health Anxiety Inventory 6
Satisfaction With Life Scale 6
36-Item Short Form Survey 6
Additional data for GAD-7, PHQ-9, PSS, SWLS and 36-SF from the iStopMM study 7
The C19Q questionnaire 7
The Symptom Impact Questionnaire 7
Height and weight 8
Vital signs 8
5-minute orthostatic test 8
Body composition 8
Grip strength 8
Smell test 8
Taste test 9
Hearing test 9
Spirometry 10
Cardiopulmonary exercise test 10
Cognitive tests 10
Digit Coding 10
Letter and Category Fluency 11
Logical Memory 11
Spatial Working Memory 11
Trail Making Test 11
Wechsler Abbreviated Scale of Intelligence 11
Blood tests 11
Ambulatory sleep study 11
Batch effect in phenotypic measures 12
Comorbidities 12
Asthma 12
Cancer 13
Coronary artery disease 13
Hypertension 13
Immunocompromised state 13
Type 2 Diabetes 13

Chronic kidney disease 14
Liver disease 14

Supplementary Figures *15*

Supplementary Figure 1. Recruitment of study participants 15

Supplementary Figure 2. Antibodies to the SARS-CoV-2 nucleocapsid (N) protein in participants 16

Supplementary Figure 3. C19 questionnaire cases and controls 17

Supplementary Figure 4. Time trends in physiological measures 18

Supplementary Figure 5. Antibody levels at time of study visit and severity of the acute infection 20

Supplementary Figure 6. Days from diagnosis of SARS-CoV-2 to study visit 21

Supplementary Figure 7. The correlation matrix for recent symptoms obtained from the C19Q questionnaire 22

Supplementary Tables *23*

Supplementary Table 1. Classification of severity of the acute phase of the SARS-CoV-2 infection 23

Supplementary Table 2. Comparing physiological test measures for individuals that participated in the deCODE health study both before and during the pandemic 24

Supplementary References *25*

Supplementary Methods

Overview of the deCODE Health Study and changes to the study to address consequences of SARS-CoV-2 infection

The deCODE Health Study (dHS)[1] is a prospective cohort study in Iceland with extensive phenotypic and genotypic information produced and collected from the participants. More than 16,000 individuals have participated in the study since its initiation in June 2016, aged between 18 and 97 years at recruitment. Participants in the dHS are invited by letter to participate in the study. The letter is followed-up with a phone call to schedule the one study visit to the deCODE Recruitment Center, in Kopavogur, Iceland, and participants are asked to complete an online questionnaire before the visit. The average duration of the study visit was 4 hours and 6 minutes. At the beginning of the visit, participants sign an informed consent, undergo a verbal interview concerning their general and current health, vital signs are taken, and potential contraindications for participation in the study as a whole or any of the individual tests are assessed. Most measures and tests in the dHS have been unchanged since the beginning of the study: height and weight, blood pressure, heart rate, pulse oximeter, temperature, bone and whole-body dual-energy X-ray absorptiometry (DXA) scan, 2D hand imaging, 3D body scan, grip strength, smell test, hearing test, voice test, visual acuity, eye endothelial imaging, ocular biomechanics, intraocular pressure, optical coherence tomography eye scan, spirometry, electrocardiogram, and cardiopulmonary exercise test (CPET). Participants also undergo several cognitive tests including letter and category fluency test, trail making test, digit symbol substitution test, rapid visual processing and spatial working memory as well as The Mini International Neuropsychiatric Interview (MINI). Blood samples are collected. A subset of participants is invited to undergo an ambulatory sleep study. In January 2018 we stopped administering the CPET to make time for additional cognitive tests: Wechsler Abbreviated Scale of Intelligence (WASI) matrix reasoning and word comprehension. When we decided to use the dHS to study the health consequences of SARS-CoV-2 infection (the dHS Covid Study), we made the following changes to the study: we modified the online questionnaire (see below), added an on-site questionnaire on SARS-CoV-2 related effects (the C19Q questionnaire), added the Wechsler logical memory test, a 5-minute orthostatic test and reinstated the CPET. To allow time for these additional tests, we removed the following tests: rapid visual processing, the bone density DXA, 2D hand imaging, 3D body scan, eye endothelial imaging, ocular biomechanics and intraocular pressure. All tests are administered by trained staff, and physicians are present at the study site.

The dHS online questionnaire on health and lifestyle

Participants in the dHS are asked to complete the dHS online questionnaire before the study visit. The dHS online questionnaire was partly changed in preparation for the dHS Covid Study including the addition of the following validated questionnaires to assess symptoms of anxiety, depression, stress, and health anxiety: General Anxiety Disorder-7 (GAD-7),[2] Patient Health Questionnaire-9 (PHQ-9)[3], Perceived Stress Scale (PSS),[4] and Short Health Anxiety Inventory (SHAI)[5] respectively, the Satisfaction With Life Scale (SWLS) and the 36-Item Short Form Survey (36-SF)[6] to assess health-related quality of life.

General Anxiety Disorder-7

The 7-item anxiety scale, GAD-7 was used to evaluate symptoms of anxiety. The GAD-7 is considered a valid and efficient tool for screening for GAD and assessing its severity in clinical practice and research.[2] In particular, evidence supports reliability and validity of the GAD-7 as a measure of anxiety in the general population.[7] The psychometric properties of the Icelandic translation of the GAD-7 have been tested and deemed satisfactory.

Patient Health Questionnaire-9

The depression module of the PHQ-9 was used to assess depressive symptoms. The PHQ-9 scores each of the 9 DSM-IV criteria for diagnosis of depression as “0” (not at all) to “3” (nearly every day). PHQ-9 has been deemed a reliable and valid measure of depression severity for use in both clinical and research settings.[3] Psychometric properties of the Icelandic PHQ-9 were adequate in primary care (Pálsdóttir, V. E. (2007). Unpublished manuscript, Department of Psychology, University of Iceland.). The Icelandic translation of PHQ-9 was used here with the permission of its authors, Agnes Agnarsdottir, Hafrún Kristjánsdóttir, Jakob Smári, Jón Friðrik Sigurðsson and Pétur Tyrfingsson.

Perceived Stress Scale

Psychological stress during the last month was assessed using the 10-item Perceived Stress Scale (PSS-10). Acceptable psychometric properties of the PSS-10 have been established in various settings.[8] Each of the 10 items are scored on a 5-point Likert scale (0-4) with a total score ranging from 0 to 40.[4] The Icelandic version of PSS-10 was used here with the permission of its author, Arna Hauksdottir. A shorter 4-item version of the Icelandic PSS has been used in studies of the effects of financial and natural disasters in Iceland.[9,10]

Short Health Anxiety Inventory

Symptoms of health anxiety were assessed using the SHAI, a 14-item version of a full length scale with comparable properties.[5] The Icelandic version of SHAI was used here with the permission of its authors, Pétur Tyrfingsson, Helgi Héðinsson and Inga Hrefna Jónsdottir. The translation was done in 2014 with the permission of Paul Salkovskis, the author of the original English version.

Satisfaction With Life Scale

Subjective overall wellbeing was evaluated with the Satisfaction With Life Scale (SWLS), a short 5-item instrument designed to measure global cognitive judgments of satisfaction with one's life.[11] Satisfactory validity and reliability of the scale have been widely tested and established.[12] The SWLS was translated to Icelandic by Daníel Ólafsson and is used here with his permission. A study of a population-based sample of individuals over 40 concluded that the Icelandic version of SWLS is a reliable and valid measure of life satisfaction (Unnarsdottir, Gisladottir, Olafsdottir, 2018).

36-Item Short Form Survey

The short form 36 health survey questionnaire (SF-36, RAND 36-Item Health Survey 1.0) was used to evaluate health related quality of life. The SF-36 is a multifactorial tool which yields an eight-scale profile of scores as well as physical and mental health summary measures.[6] It is a generic measure that does not target a specific age, disease or treatment group, and has been validated for use among various patient groups and for the general population.[13,14] The Icelandic SF-36 questionnaire used for this study is based on a translation from

GlaxoSmithKline, with minor changes. The original translation was done according to the standards of the International Quality of Life Assessment (IQOLA) project.[15] We estimated the correlated physical health (PCSc) and mental health (MCSc) scores and uncorrelated scores; PCSuc and MCSuc.[16]

Additional data for GAD-7, PHQ-9, PSS, SWLS and 36-SF from the iStopMM study

For additional analysis of GAD-7, PHQ-9, PSS, SWLS and 36-SF scores, we obtained data from the "Iceland Screens, Treats, or Prevents Multiple Myeloma" (iStopMM) study (ClinicalTrials.gov Identifier: NCT03327597). The iStopMM study is a population-based screening study for monoclonal gammopathy of undetermined significance (MGUS) and a randomized controlled trial of follow-up strategies that began in 2016. All 148,704 individuals in Iceland born in 1975 or earlier received a letter inviting them to participate. Participation was based on informed consent and data are stored using encrypted identifiers. A total of 80,759 (54%) provided informed consent to participate in the study. While the main outcome measure of iStopMM is the effect of early detection and treatment of multiple myeloma precursor on survival, a secondary goal is to understand the mental health effects associated with screening an entire population for a cancer precursor. Beginning in 2018 nearly sixty thousand participants who provided their email address have received questionnaires annually, excluding those patients who have been randomized for follow-up in iStopMM. Through iStopMM we thus obtained additional contemporary control data aquired during the pandemic, as well as historical data (before February 2020) for a subset of cases and contemporary controls, allowing for comparison of measures before and after the SARS-CoV-2 infection for cases, and similar analysis of longitudinal measure for controls.

The C19Q questionnaire

Participants answered a self-administered questionnaire on SARS-CoV-2 related effects (the C19Q) on a tablet computer at the study site. The questionnaire was designed by the study authors in Icelandic and has not been formally translated to other languages.

In addition to administering the C19Q in the dHS Covid Study starting in September 2020, we invited a random sample, matched with SARS-CoV-2 cases for age and sex, of pre-pandemic participants (historical controls) in the dHS to answer it online after September 2020 (additional C19Q controls). As of this analysis, 760 pre-pandemic participants (historical controls) in the dHS have answered the questionnaire between September 2020 and November 2021.

The Symptom Impact Questionnaire

The Symptom Impact Questionnaire (SIQR) was added to the C19Q after the study began, in November 2021, to assess symptoms of fatigue. The SIQR was translated to Icelandic by Gunnar Tómasson and Arnór Víkingsson with the permission of Robert Bennett and is used here with their permission and the permission of the Mapi Research Trust, provided by Ronald Friend. The translation was done using the FACIT-methodology that involves i) 2 independent forward translations by native Icelanders with excellent skills in English and insight into health measurement scales, ii) consolidation to a single Icelandic translation, iii) backward translation of the consolidated Icelandic version by a native English speaker iv) comparison of the original instrument in English and the back-translated version by 2 experienced clinical investigators with expertise in development and

validation of health measurement scales and v) fine-tuning of the Icelandic translated instrument based on comments obtained in step iv).

Height and weight

Height and weight were measured with the seca 285 electronic measuring station (seca, Germany). Body mass index (BMI) was calculated in the standard manner, BMI = kg/m^2. Obesity was defined as BMI ≥ 30.

Vital signs

Resting blood pressure was measured according to guidelines[17] using the SureSign Vs4 equipment (CIGA Healthcare Ltd, UK). The blood pressure was measured in the left arm unless contraindications were present, with the participant seated, using the appropriate size cuff and positioning the cuff at the level of the patient's right atrium. The SureSign Vs4 equipment was also used to measure resting heart rate and oxygen saturation of peripheral arterial blood with a pulse oximeter.

5-minute orthostatic test

The 5-minute orthostatic test was administered directly following the electrocardiogram test. Supine blood pressure and heart rate were recorded after relaxation. Participant was then directed to stand up with their shoulders leaning against a wall and heels approximately 15 cm from the wall. Blood pressure and heart rate were immediately recorded and then again, every minute for 5 minutes, for a total of 6 recordings while standing. We used the SureSign Vs4 equipment (CIGA Healthcare Ltd, UK). Symptoms during test were recorded. The blood pressure and heart rate differences were calculated as the measures after three and five minutes subtracting the measures taken when lying down. Orthostatic hypotension (OH) cases were defined as those with a fall of >20 mmHg systolic or >10 mmHg diastolic after standing for three minutes. Postural orthostatic tachycardia (POTS) cases were defined as those with heart rate increase > 30 bpm after standing for 5 minutes.

Body composition

Body composition (distribution of lean and fat mass) was assessed using whole-body fan beam dual-energy X-ray absorptiometry (DXA) scan (Horizon QDR series System, S/N200547, Hologic Discovery A, Hologic Inc., Bedford, USA). The DXA equipment was calibrated daily and scans were performed by trained technicians according to manufacturer instructions. The measures that we used from the scan in our analyses are lean mass index (LMI) and fat mass index (FMI).

Grip strength

Grip strength was measured in a standardized manner based on published recommendations using the JAMAR Hydraulic Hand Dynamometer[18]. Three measurements were performed for each hand and hand dominance was recorded. The maximum grip strength of the 6 measures was used for the analyses.

Smell test

Odor perception was tested using six odor pens from Sniffin' Sticks (Burghart Messtechnik GmbH, Wedel, Germany); licorice, cinnamon, fish, lemon, peppermint, and banana.[20,21] The experimenter placed the odor pen approximately two cm in front of participants' nose for three seconds. Participants were first asked to name the

smell (odor naming task). Participants then provided an intensity rating on a Likert scale from one to seven (very mild to very strong), followed by a pleasantness rating from one to seven (very unpleasant to very pleasant). A minimum of 30 seconds passed between presentations of each odor. Following the smell test, the participants answered questions regarding symptoms of abnormal olfactory function (introduced in the dHS for the dHS Covid Study).

Odor intensity and pleasantness ratings for all six odors were summed to form an aggregate odor intensity scale and odor pleasantness scale (individuals who reported that they could not smell the odor at all were assigned zero points in the intensity rating for that odor). We identified hyposmia cases based on a cutoff at the 10th percentile (per sex and 10 year age groups) of the odor intensity scale in a sample of 13,863 Icelanders before the pandemic, in line with previous approaches that have used the 10th percentile as a cutoff for hyposmia.[21,22] We subsequently used this threshold to define hyposmia cases in our complete dataset. We identified selective anosmia cases as those who reported finding no smell for at least one odor. Complete anosmia cases, defined as those who found no smell for at least five odors, were too few to include in the analysis. Responses in the odor naming task were coded as correct (two points), near-misses (one point) and far-misses (zero points) as previously described[19]. An identification score was computed by summing the odor naming points for all 6 odors. Identification of individual odors was defined as correct naming (two points).

More detailed description of the smell test used in the dHS can be found in ref[19].

Taste test

The taste test was introduced in the dHS for the dHS Covid Study. We used taste solutions for whole mouth suprathreshold testing of gustatory function (sweet 1 g sucrose in 10 g aqua; sour 0.5 g citric acid in 10 g aqua; salty 0.75 g sodium chloride in 10 g aqua; bitter 0.005 g quinine hydrochloride in 10 g aqua).[23,24] For each tastant, participants were given a new plastic spoon with 2 drops of taste solution and asked to identify the taste from a multiple-choice list (sweet, sour, salty, bitter). Participants then provided an intensity rating of the taste from one to seven (very weak to very strong) and pleasantness rating from one to seven (very unpleasant to very pleasant). After each taste participants rinsed their mouth. Following the taste test, participants answered questions regarding symptoms of abnormal gustatory function.

Hypogeusia cases were defined as correct identification of two or fewer tastes. Selective ageusia cases were defined as those who described that they found no taste for at least one taste solution. Complete ageusia cases, defined as those who found no taste for at least three taste solutions, were too few to include in the analysis. Taste intensity and pleasantness ratings for all four tastes were summed to form an aggregate taste intensity scale and taste pleasantness scale (individuals who reported not tasting the taste at all were assigned 0 points in the intensity rating for that taste).

Hearing test

The hearing test in the dHS has been described in detail. Pure tone air conduction audiometric screening test was performed by trained staff using the AS608e audiometer (Interacoustics, Denmark). The audiometer delivers pure tones at 0.5, 1, 2, 4, 6 and 8 kHz at different intensity levels, usually starting at 20 dB HL and increased if necessary. For each individual and each ear, the lowest intensity of sound detection is defined as their hearing

threshold at that frequency. The pure tone average (PTA) was defined as the average hearing threshold at 0.5, 1, 2 and 4 kHz (according to the WHO classification.[29] We define PTA hearing impairment cases as those with PTA>25, and hearing impairment at each frequency, at 0.5, 1, 2, 4, 6 and 8 kHz, was defined as those with hearing thresholds at the corresponding frequency >25. More detailed description of the hearing test used in the dHS can be found in ref[25].

Spirometry

Post-bronchodilator spirometry was performed using the EasyOne Pro equipment from Medical Technologies in a standardized manner and following published guidelines.[30] Two different machines were used in the study. Albuterol was used for bronchodilation. The measures from the spirometry used for the analyses were lower forced expiratory volume in 1 second (FEV1), forced vital capacity (FVC) and the FEV1/FVC ratio.

Cardiopulmonary exercise test

Participants were tested on a cycle ergometer using an incremental ramp protocol in a standardized manner following published guidelines[28,29]. Based on a preparation pilot trial of 100 healthy volunteers, we estimated participant workload using the Wasserman equation[30] (accounting for sex, height and weight) and multiplying the result by 1.33. We used three Jaeger Vyntus CPX instruments coupled with SentrySuite software for the study. Gas exchange and ventilatory variables were measured continuously as the subjects breathed into a two-way breathing mask that was individually fitted and leak tested. The gas analyzers used were volume- and gas calibrated for barometric pressure, temperature and humidity after each test. Blood pressure was measured every two minutes with a standard cuff sphygmomanometer. Electrocardiogram was obtained at rest in supine position prior to the test and continuously during the test. Oxygen haemoglobin saturation was monitored continuously with a pulse oxymeter.

The test started after the bike has been fitted to the participant and breathing mask, electrocardiogram leads, sphygmomanometer and pulse oximeter connected. The test had four phases: 1) a two minute pre-exercise phase during which the participant rested on the bike and baseline parameters were collected, 2) a one minute warm-up phase without resistance, 3) the exercise phase which lasted on average 10:41 (± 2:27) minutes for women and 9:16 (± 1:47) minutes for men, and 4) a two minute post-exercise phase during which the participant recovered on the bike and post-exercise parameters were collected.

In preparation for the test participants were asked to avoid heavy meals within three hours of testing, consumption of alcohol within 12 hours of testing, other vigorous physical exercise within 16 hours, wearing restricting clothing during the test and smoking within three hours of testing.

The measures from the cardiopulmonary exercise test used in the analyses were load and VO2 max.

Cognitive tests

Digit Coding

The Coding subtest[31] from the Wechsler Adult Intelligence Scale, fourth edition, was administered, where participants were asked, using a specific key where symbols are paired with numbers, to correctly draw as many symbols as they can within a 120 second time limit. The number of correctly drawn symbols was recorded.

Letter and Category Fluency

The participants were asked to name as many words as they could in one minute, either starting with a certain letter (two attempts, S and H, Letter Fluency[36]) or a part of a certain semantic category (i.e. animals, Category Fluency[37]). The total number of words named for both letters was used as an outcome measure of Letter Fluency, and the total number of animals named was used as an outcome for Category Fluency. More detailed description of these tests can be found in ref[32].

Logical Memory

Memory was assessed using the Logical Memory (LM) subtest[32,35] from Wechsler Memory Scale, third edition. In brief, short stories were read to the participants and the participants subsequently asked to freely recall as much as they could from the stories, both immediately (LM-I Recall Score)) or after a 20-minute delay (LM-II Recall Score). Following the delayed free recall, participants were also asked yes-or-no questions form the stories (LM-II Recognition Score). The LM test was added to the dHS for the Covid Study and thus only contemporary controls were available for comparison between cases and controls.

Spatial Working Memory

Spatial Working Memory (SWM)[39] is part of the Cambridge Neuropsychological Test Automated Battery (CANTAB) and has been described previously.[32] Participants are presented with boxes on the screen and are instructed to find tokens hidden under them, one at a time. Participants need to remember where they have already searched and where they have already found tokens. Outcome measures used were average between-search errors (i.e. errors where participants searched boxes where they had already found tokens) on screens containing eight boxes (SWM Between-Search Errors 8) and strategy score (SWM Strategy Score), an indicator of errors in the use of a consistent strategy.

Trail Making Test

A paper-and pencil version of the Trail Making Test (TMT)[37] was administered to participants, where they were asked to connect numbers from one through 25 (TMT-A) or to connect numbers and letter, alternating between the two (TMT-B). The time it took participants to complete these tasks was recorded. More detailed description of these tests can be found in ref[32]

Wechsler Abbreviated Scale of Intelligence

Two subtests, Matrix Reasoning and Vocabulary, of the Icelandic version of the Wechsler Abbreviated Scale of Intelligence (WASI-IS)[38,39] were administered, and the recorded scores used directly as outcome measures.

Blood tests

For blood measurements we used the Sysmex XN-1000 hematology analyzer (Sysmex Corporation, Japan) and the cobas 6000 chemistry analyzer (Roche Diagnostics, Switzerland) according to manufacturer's instructions, using reagents from the relevant manufacturer.

Ambulatory sleep study

A subset of participants in the dHS study are invited to participate in a Self Applied Somnogram study using the modified A1 System from Nox Medical. Here we report heart rate and heart rate variability (HRV) during sleep. R peak detection was used to located heart beats to estimate heart rate and heart rate variability from electrocardiogram (ECG) sleep measurements. To remove false positive beats signal quality indices were

calculated.[40] K-means clustering was used to split the ECG recordings into wake and sleep states. The wake state was not used for analysis, as it includes device setup artifacts. Heart rate was estimated by calculating the mean elapsed time between successive heart beats and HRV was estimated using the root mean square of successive differences (RMSSD) between heartbeats.

Batch effect in phenotypic measures

The phenotypic measures were collected both before the pandemic (historical controls), and during the pandemic (contemporary controls). This data availability during the time from June 2016 to November 2021 allowed for batch effects in physiological, blood, and cognitive measures. We compared measures between SARS-CoV-2 cases and controls, using all available controls and contemporary controls separately. Additionally, we compared measures between historical and contemporary controls, where same direction of effect for contemporary controls as for cases was interpreted as a possible batch effect in the outcome measures. We then plotted the distribution of the measures by months during the data collection period to help characterize possible batch effects (Supplementary Figure 7). From these analyses, we observed batch effects for the hearing test, oxygen saturation, grip strength and blood tests.

As an example, for the hearing test, the hearing thresholds were lower for cases compared to all controls and also for contemporary controls compared to historical controls. Plotting pure tone average (PTA) over time we observed that hearing thresholds were higher during a time period from 2018 to 2019 (Supplementary Figure 7.a). Therefore, we only used controls measured after 2019 for the hearing test.

Grip strength measures were lower during a time period in 2017 (Supplementary Figure 7.c). Therefore, we only used controls measured after 2017 for the grip strength test. For the oxygen saturation test, we observed that SpO2 was higher after 2019 (Supplementary Figure 7.b). Therefore, we only used contemporary controls for the oxygen saturation test. Due to batch effects detected in a similar vein as described above, we restrict the control dataset to only contemporary controls for oxygen saturation test (SpO2) (Supplementary Figure 7.b.).

Because of observed volatility in the blood test measures, often during the whole data collection period and hence, the need to adjust for time effects, we only compare blood measures of cases with those of contemporary controls and adjust for time by applying a smoothing function to month of measure indicator using generalized additive model (gam() and s() in R mcgv package with smooth class = thin plate regression splines). The results were robust to changing the functional form of time in the blood equations to month or week indicators.

Comorbidities

Asthma

Asthma diagnoses were based on a combination of physician's diagnoses and/or ICD-10 diagnoses, including anyone of J45.0, J45.1, J45.8, J45.9, and J46 and/or self-reported by a positive reply to the question: has a doctor confirmed your asthma diagnosis.[42] The data are derived from Landspitali - The National University Hospital of Iceland, Laeknasetrid Medical Center, as well as Register of Contacts with Medical Specialists in Private Practice and those that had received two or more asthma diagnosis in the Register of Primary Health Care Contacts. Data was collected from the years 1977 to 2020.

Cancer

Information on cancer in the study population comes from the Icelandic Cancer Registry, which registers all cancer diagnoses in the population using the ICD system. The registry was used to identify cancer diagnoses in the study subjects, including ICD-10 codes C00–C96 with the exception of ICD-10 C44 (non-melanoma skin cancer).[43]

Coronary artery disease

Coronary artery disease diagnoses were based on discharge diagnoses from Landspitali – The National University Hospital of Iceland, the only tertiary care center in Iceland, and data from the Icelandic Causes of Death Register, as previously described.[44] Coronary artery diseases case status was assigned based on ICD-10 codes (or comparable ICD-9 codes) indicative of coronary artery disease (ICD-10: I20.0, I21.x, I22.x, I24.x, or I25.x). Data were collected from 1987 to 2020.

Hypertension

Persons with hypertension were identified as those that had been given the diagnosis by ICD code and those that had used antihypertensives continuously for more than 1 year (C02*). Information about hypertension diagnoses were obtained from Landspitali - the National University Hospital of Iceland in Reykjavik, the Primary Health Care Clinics of the Reykjavik area, the Register of Contacts with Medical Specialists in Private Practice and the Register of Primary Health Care Contacts both kept by the Directorate of health, using ICD-10 code I10 and ICD-9 code 401. Data was collected from 1987 to 2020. The drug data originates from the Prescription Medicines Register organized and maintained by the Directorate of Health since 2001.

Immunocompromised state

Immunocompromosed state was defined as immunocompromised from solid organ transplant, blood or bone marrow transplant, immune deficiencies, HIV, use of corticosteroids, or use of other immune weakening medicines. The phenotype was based on the following ICD-10 codes (and comparable ICD-9 codes): Z94.0, Z94.1, Z94.2, Z94.3, Z94.4, Z94.82, Z94.83, Z94.81, Z94.84, B20, D80, D81, D82, D83, D84, D86 or the use of the following drugs: H02A* corticosteroids for systemic use, plain, H02B* corticosteroids for systemic use, combinations, L04A* immunosuppresants, requiring two prescriptions in the two years prior to study participation. The ICD codes originated from Landspitali - the National University Hospital of Iceland in Reykjavik, the Register of Contacts with Medical Specialists in Private Practice and the Register of Primary Health Care Contacts in Iceland. The drug data originates from Landspitali - the National University Hospital of Iceland and the Prescription Medicines Register organized and maintained by the Directorate of Health.

Type 2 Diabetes

Type 2 diabetes cases were identified based on ICD-10 code E11, use of oral diabetes medication (ATC A10B), HbA1C > 6.5, or self-reported history of type 2 diabetes. Individuals with type 1 diabetes (ICD-10 E10) were excluded as cases.[45] The ICD codes originate from the Landspitali – The National University Hospital of Iceland, and the Register of Contacts with Medical Specialists in Private Practice and the Register of Primary Health Care Contacts, both kept by the Directorate of Health. The drug data originates from the Prescription Medicines Register organized and maintained by the Directorate of Health since 2001.

Chronic kidney disease

Chronic kidney disease was defined as ICD code for stage 3-5 chronic kidney disease (ICD-10 N18.3-5 and comparable ICD-9 code) or estimated glomerular filtration rate (eGFR) <60 ml/min/1.73 m^2 as for at least three months.[46,47] When defining chronic kidney disease, all serum creatinine measurements obtained during an episode of acute kidney injury were excluded and individuals who had eGFR <60 ml/min/1.73 m^2 for <3 months duration were excluded from the chronic kidney disease sample set. The ICD codes originated mainly from Landspitali - the National University Hospital of Iceland in Reykjavik.

Liver disease

We used the following ICD codes to define diseases of liver: ICD-10 K70-77 and ICD-9 570-573. The ICD codes originate from the Landspitali – The National University Hospital of Iceland, and the Register of Contacts with Medical Specialists in Private Practice and the Register of Primary Health Care Contacts, both kept by the Directorate of Health.

Supplementary Figures

Supplementary Figure 1. Recruitment of study participants

The flowchart shows how cases and controls were defined in our study.

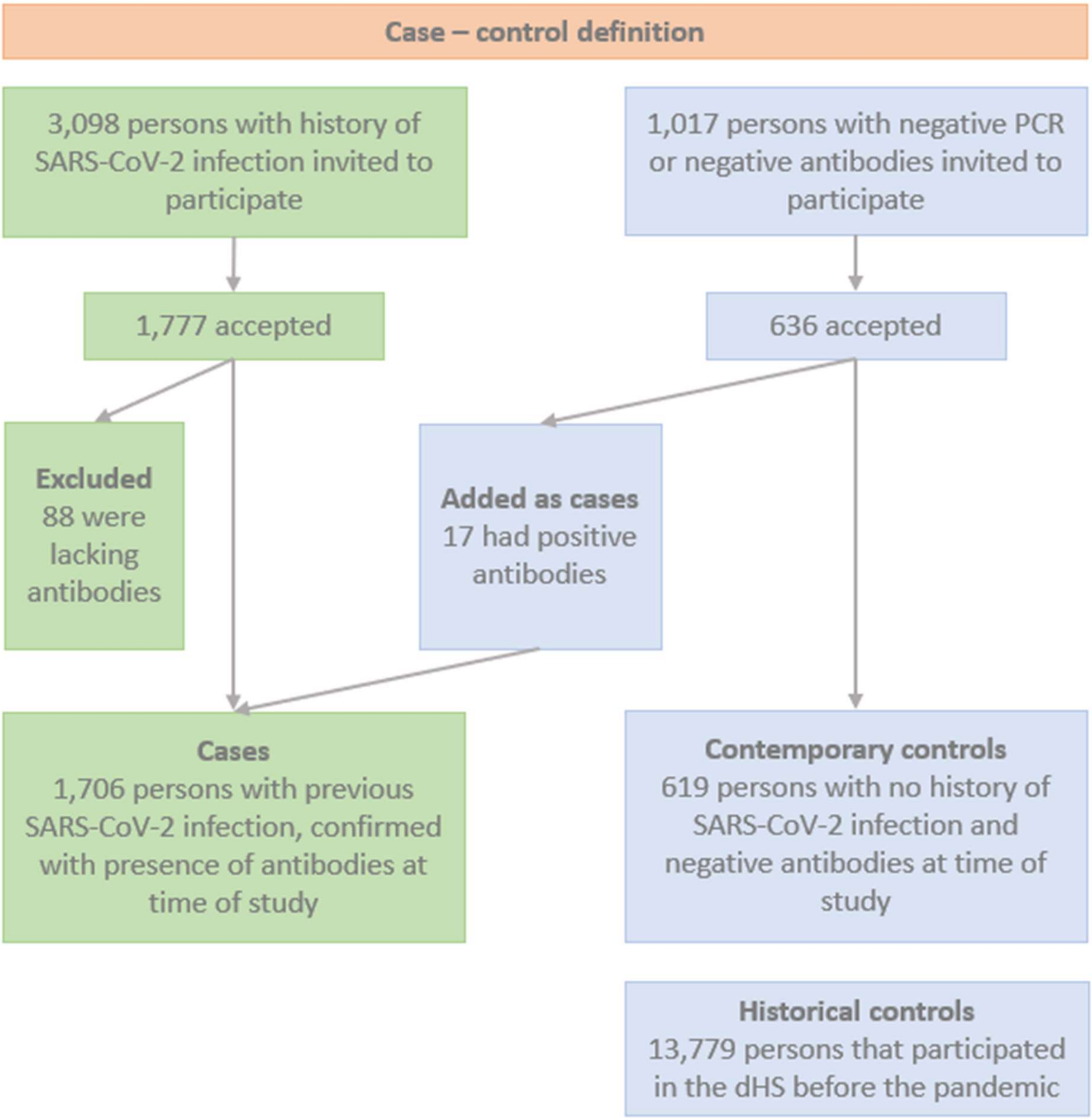

Supplementary Figure 2. Antibodies to the SARS-CoV-2 nucleocapsid (N) protein in participants

The figure shows a histogram of the levels of antibodies to the nucleocapsid (anti-N) protein on the log scale, for individuals with history of infection (n=1,777) and individuals with no history of infection (n=636). The manufacturer recommended threshold for positive antibodies is 1 (0 on the log scale). The 17 individuals without history of infection but anti-N antibodies >1 were defined as cases. The 88 individuals with history of infection but antibodies <0.2 (-1.6 on the log scale) were excluded from the analysis. The grey dotted lines show the thresholds used.

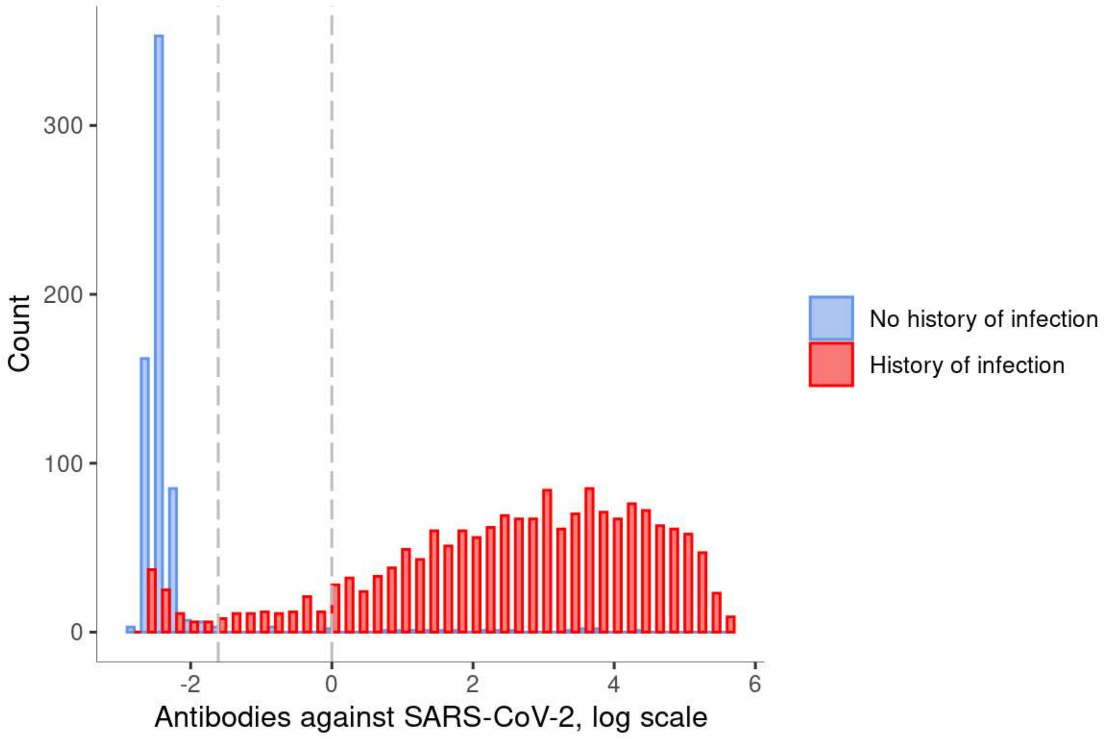

Supplementary Figure 3. C19 questionnaire cases and controls

The definition of C19Q cases and controls.

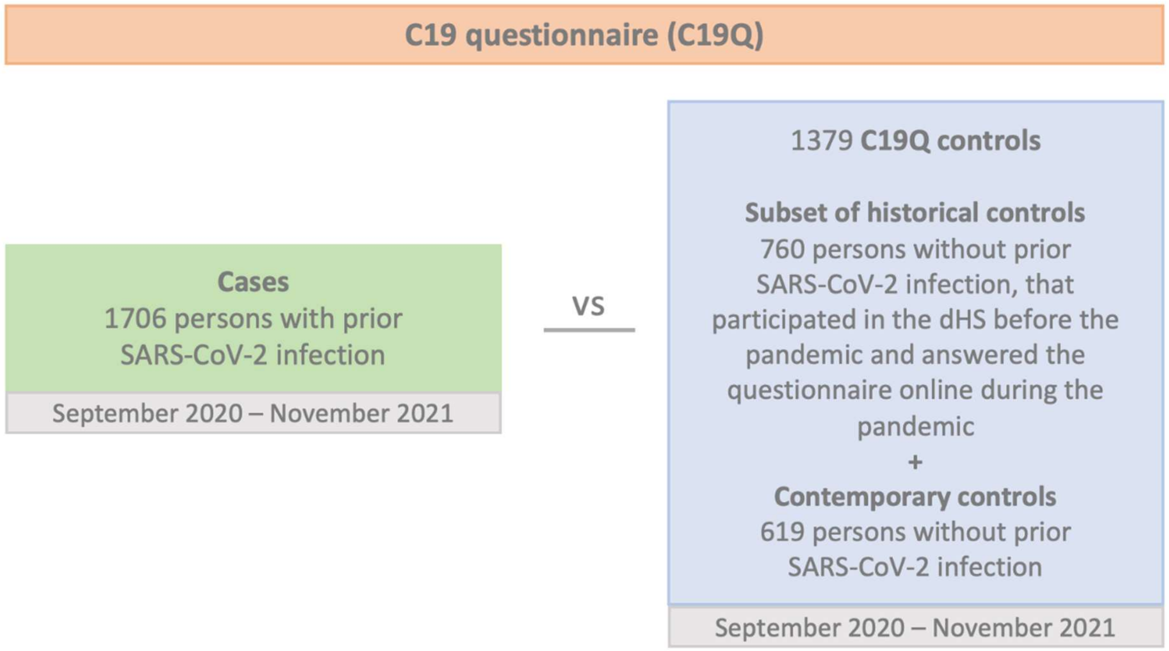

Supplementary Figure 4. Time trends in physiological measures

The figures show boxplots for each month during the time the measurements were collected, for all historical controls (n=13,779). Time trends were observed for **a)** pure tone average (PTA) hearing thresholds from the hearing test, **b)** oxygen saturation (SpO2), **c)** grip strength. The box plots show the median levels (horizontal lines), interquartile range (IQR) (box) and the whiskers extend to the most extreme data points within a distance of 1.5 times the IQR from the edges of the box.

a)

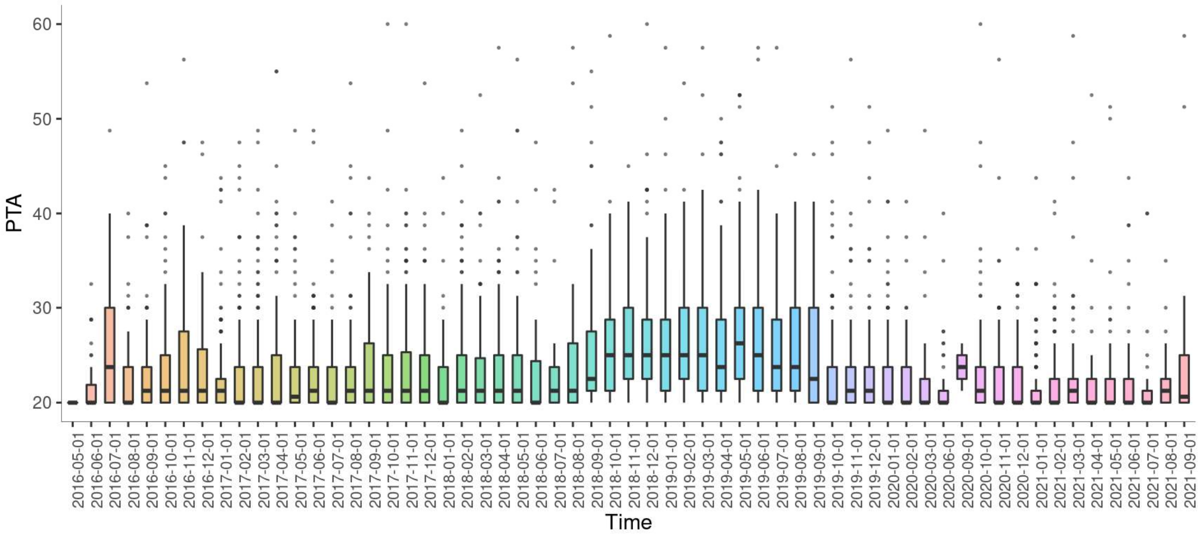

b)

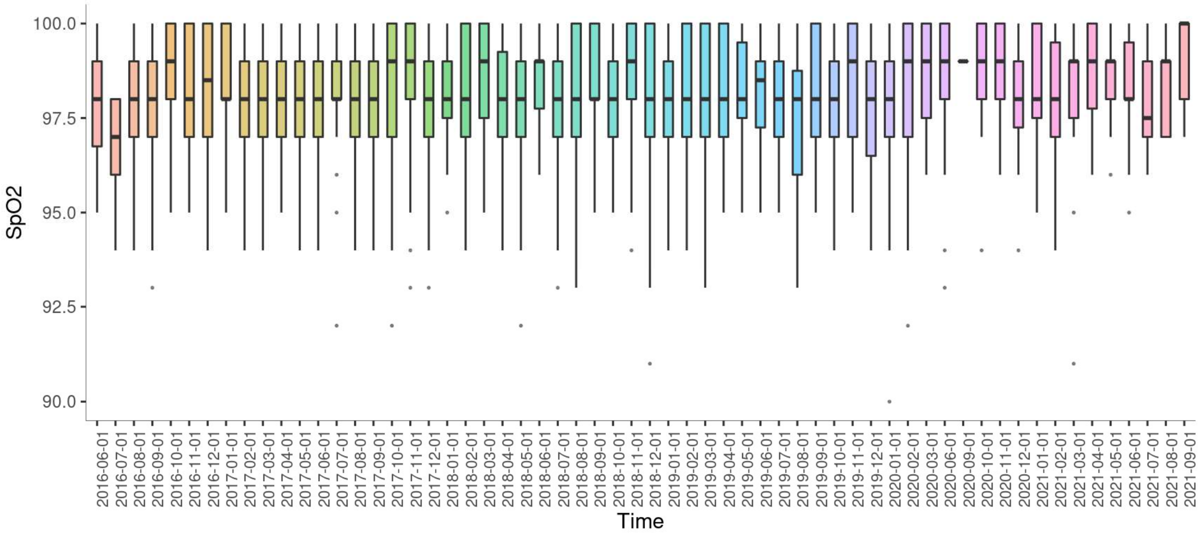

c)

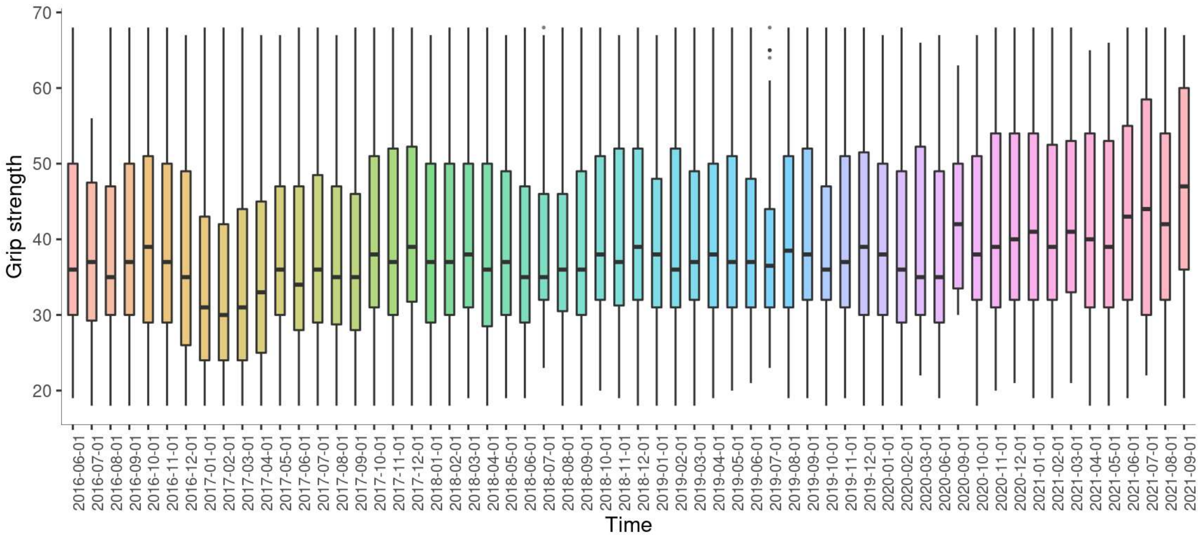

Grip strength
70
60
50
40
30
20
2016-06-01
2016-07-01
2016-08-01
2016-09-01
2016-10-01
2016-11-01
2016-12-01
2017-01-01
2017-02-01
2017-03-01
2017-04-01
2017-05-01
2017-06-01
2017-07-01
2017-08-01
2017-09-01
2017-10-01
2017-11-01
2017-12-01
2018-01-01
2018-02-01
2018-03-01
2018-04-01
2018-05-01
2018-06-01
2018-07-01
2018-08-01
2018-09-01
2018-10-01
2018-11-01
2018-12-01
2019-01-01
2019-02-01
2019-03-01
2019-04-01
2019-05-01
2019-06-01
2019-07-01
2019-08-01
2019-09-01
2019-10-01
2019-11-01
2019-12-01
2020-01-01
2020-02-01
2020-03-01
2020-06-01
2020-09-01
2020-10-01
2020-11-01
2020-12-01
2021-01-01
2021-02-01
2021-03-01
2021-04-01
2021-05-01
2021-06-01
2021-07-01
2021-08-01
2021-09-01
Time

Supplementary Figure 5. Antibody levels at time of study visit and severity of the acute infection

The figure shows boxplots for antibodies to the SARS-CoV-2 nucleocapsid protein among individuals in each severity category. The number of individuals in each category is; N=678, N=466, N=464 and N=85, for categories 1,2,3 and 4, respectively. The box plots show the median levels (horizontal lines), interquartile range (IQR) (box) and the whiskers extend to the most extreme data points within a distance of 1.5 times the IQR from the edges of the box.

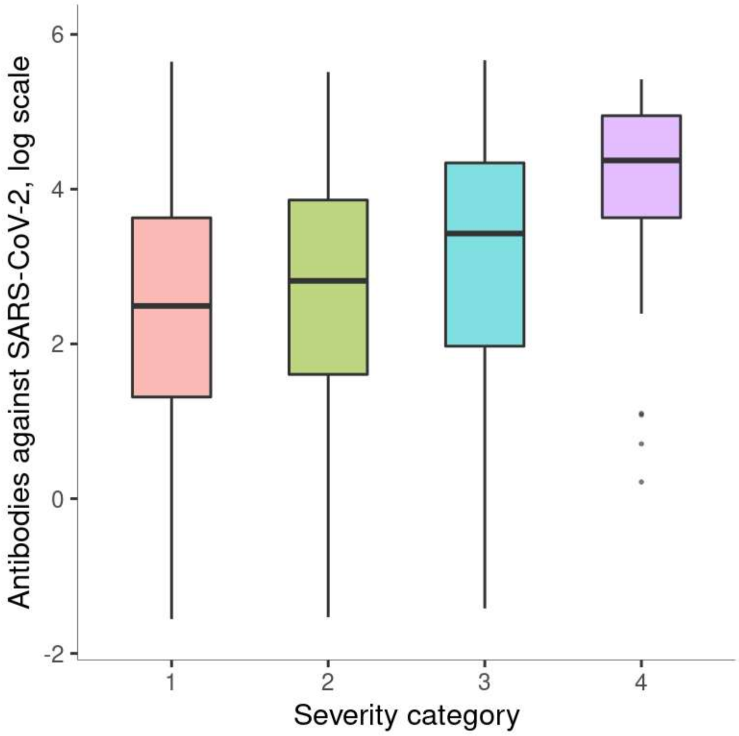

Supplementary Figure 6. Days from diagnosis of SARS-CoV-2 to study visit

A histogram that shows the distribution of days from diagnosis for the SARS-CoV-2 cases participants (n=1,706).

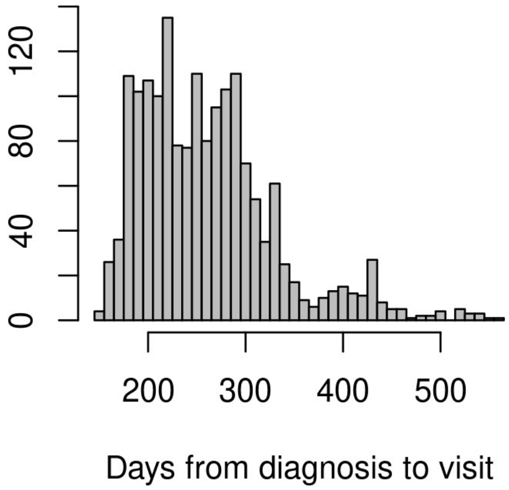

Supplementary Figure 7. The correlation matrix for recent symptoms obtained from the C19Q questionnaire

The correlation matrix is shown for the symptoms that associate most significantly with SARS-CoV-2 status ($P < 1.0 \times 10^{-6}$). The symptoms are ordered using a hierarchical clustering order. These are symptoms that the participants were asked about at time of study and they were asked to consider the symptoms they had experienced during the four weeks prior to study visit. For cases, those that had contracted SARS-CoV-2, the symptoms were assessed five to 18 months after the acute infection.

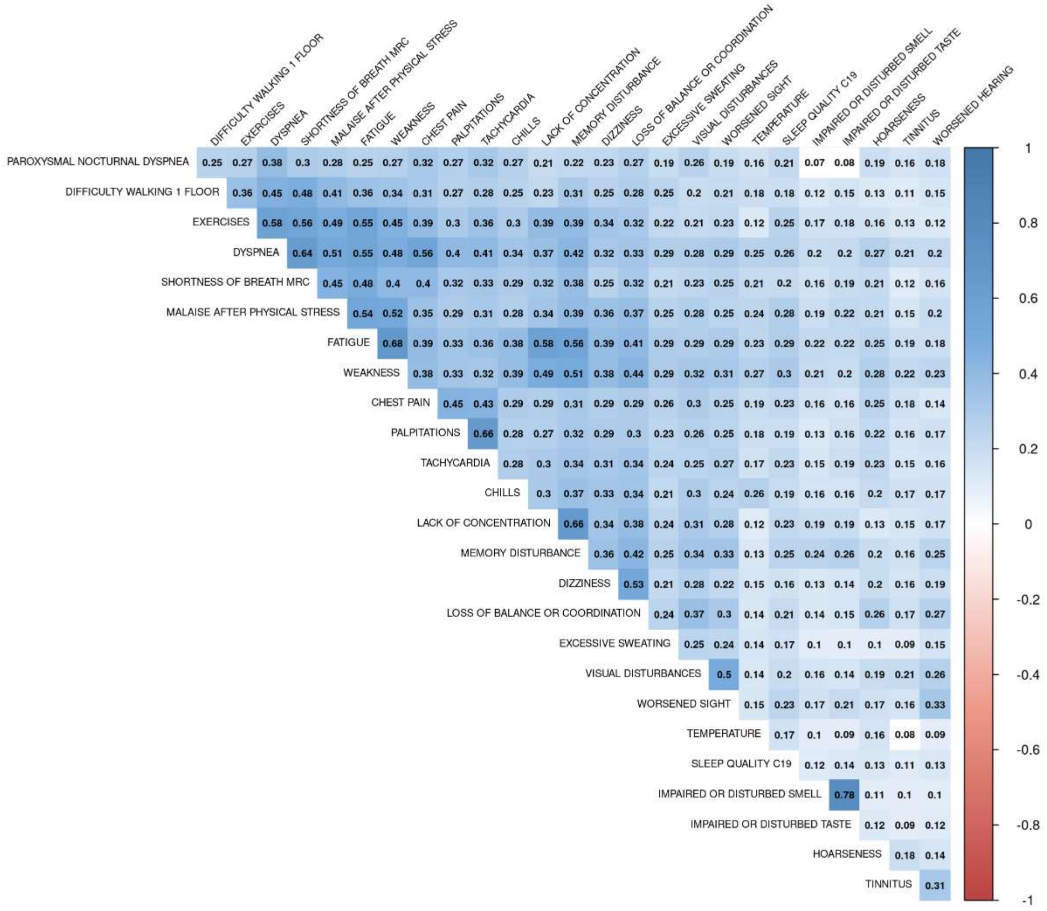

Supplementary Tables

Supplementary Table 1. Classification of severity of the acute phase of the SARS-CoV-2 infection

We have scaled the severity of the acute infection based on intensity of treatment, severity assessment by the Telehealth monitoring service of the Covid-19 outpatient clinic at Landspitali – the National University Hospital (LUH) in Reykjavik, Iceland, and self-assessment. Severity scale ranges from 0 (least severe acute infection) to 8 (most severe acute infection). To simplify downstream analyses, we have grouped the severity scales into categories such that two groups from the severity scale are in each category and 1 equals least severe acute infection and 4 most severe acute infection.

Severity scale	N (%)	N women (%)	N men (%)	Severity category	N (%)	Self-assessment of severity of illness	Severity assessment by the Telehealth monitoring service	Intensity of treatment
1	218 (13.1%)	100 (11.9%)	118 (14.4%)	1	678 (40.8%)	No or very mild illness	Low	No physical assessment at LUH
2	460 (27.7%)	221 (26.2%)	239 (29.1%)			Mild	Low	No physical assessment at LUH
3	323 (19.4%)	171 (20.3%)	152 (18.5%)	2	466 (28.0%)	Moderate	Low	No physical assessment at LUH
4	143 (8.6%)	62 (7.4%)	81 (9.9%)			Severe or very severe	Low	No physical assessment at LUH
5	283 (17.0%)	156 (18.5%)	127 (15.5%)	3	434 (26.1%)	NA	Moderate/high	No physical assessment at LUH
6	151 (9.1%)	100 (11.9%)	51 (6.2%)			NA	NA	Evaluation at the Covid-19 clinic
7	66 (4.0%)	29 (3.4%)	37 (4.5%)	4	85 (5.1%)	NA	NA	Hospitalization
8	19 (1.1%)	4 (0.5%)	15 (1.8%)			NA	NA	Hospitalization ICU

ICU=Intensive care unit

Supplementary Table 2. Comparing physiological test measures for individuals that participated in the deCODE health study both before and during the pandemic

The effect of SARS-CoV-2 status on the difference between pandemic and pre-pandemic measurements, adjusting for age, sex and time between measures (linear regression). CI=95% confidence interval.

**Test**	**Measure**	**N cases**	**N control**	**Effect**	**CI lower**	**CI upper**	**P-value**
Smell	Odor intensity scale	122	294	-2.24	-3.30	-1.18	3.3E-05
Smell	Odor intensity cinnamon	122	294	-0.58	-0.87	-0.29	9.0E-05
Smell	Odor intensity licorice	122	294	-0.56	-0.88	-0.25	5.2E-04
Smell	Odor intensity lemon	122	294	-0.56	-0.89	-0.24	6.5E-04
Smell	Odor intensity banana	122	294	-0.30	-0.60	0.00	4.9E-02
Smell	Odor intensity peppermint	122	294	-0.22	-0.47	0.02	7.4E-02
Spirometry	FEV1	115	289	-0.86	-2.00	0.27	1.4E-01
Smell	Odor pleasantness licorice	122	294	-0.17	-0.43	0.08	1.7E-01
CPET	VO2 max	76	173	-0.89	-2.29	0.51	2.1E-01
Hearing	Hearing threshold at 1 kHz	39	66	-2.87	-7.61	1.88	2.4E-01
DXA	BMI	121	280	-0.20	-0.58	0.17	2.9E-01
Smell	Odor pleasantness lemon	122	294	-0.15	-0.43	0.13	3.0E-01
Spirometry	FEV1/FVC ratio	115	289	0.00	-0.01	0.00	3.0E-01
Hearing	Hearing threshold at 8 kHz	39	66	3.73	-3.45	10.90	3.1E-01
Vital signs	Heart rate	118	288	-0.84	-2.72	1.05	3.8E-01
Smell	Odor pleasantness cinnamon	121	294	-0.11	-0.38	0.15	4.1E-01
DXA	FMI	121	280	-0.21	-0.72	0.29	4.1E-01
Vital signs	SpO2	118	288	0.92	-1.44	3.28	4.4E-01
Smell	Odor pleasantness banana	122	294	-0.11	-0.38	0.17	4.5E-01
Hearing	Hearing threshold at 2 kHz	39	66	-2.37	-8.71	3.97	4.6E-01
Spirometry	FVC	115	289	-0.38	-1.45	0.70	4.9E-01
Hearing	PTA hearing threshold	39	66	-1.63	-6.33	3.08	5.0E-01
Smell	Odor pleasantness scale	120	294	-0.29	-1.16	0.57	5.1E-01
Smell	Identification score	120	294	-0.16	-0.73	0.41	5.8E-01
Smell	Odor pleasantness fish	121	294	0.08	-0.20	0.36	5.8E-01
Smell	Odor pleasantness peppermint	122	294	-0.06	-0.28	0.16	6.0E-01
Grip strength	Grip strength	59	139	0.45	-1.50	2.40	6.5E-01
Grip strength	Grip strength men	23	60	0.39	-1.81	2.59	7.3E-01
Hearing	Hearing threshold at 0.5 kHz	39	66	-0.46	-3.24	2.32	7.5E-01
Hearing	Hearing threshold at 4 kHz	39	66	-1.06	-8.44	6.33	7.8E-01
CPET	Load	77	175	-0.87	-7.21	5.47	7.9E-01
DXA	LMI	121	280	-0.02	-0.22	0.17	8.1E-01
Hearing	Hearing threshold at 6 kHz	39	66	0.42	-6.77	7.61	9.1E-01
Smell	Odor intensity fish	122	294	-0.01	-0.27	0.25	9.4E-01
Vital signs	Diastolic blood pressure	118	288	0.01	-0.28	0.30	9.4E-01
Grip strength	Grip strength women	36	79	0.12	-3.38	3.62	9.5E-01
Vital signs	Systolic blood pressure	118	288	-0.04	-2.84	2.77	9.8E-01

Supplementary References

1. Ivarsdottir, E. V. *et al.* Sequence variation at ANAPC1 accounts for 24% of the variability in corneal endothelial cell density. *Nat. Commun.* **10**, 1284 (2019).
2. Spitzer, R. L., Kroenke, K., Williams, J. B. W. & Löwe, B. A brief measure for assessing generalized anxiety disorder: the GAD-7. *Arch. Intern. Med.* **166**, 1092–1097 (2006).
3. Kroenke, K., Spitzer, R. L. & Williams, J. B. The PHQ-9: validity of a brief depression severity measure. *J. Gen. Intern. Med.* **16**, 606–613 (2001).
4. Cohen, S., Kamarck, T. & Mermelstein, R. A global measure of perceived stress. *J. Health Soc. Behav.* **24**, 385–396 (1983).
5. Salkovskis, P. M., Rimes, K. A., Warwick, H. M. C. & Clark, D. M. The Health Anxiety Inventory: development and validation of scales for the measurement of health anxiety and hypochondriasis. *Psychol. Med.* **32**, 843–853 (2002).
6. Ware, J. E. & Sherbourne, C. D. The MOS 36-item short-form health survey (SF-36). I. Conceptual framework and item selection. *Med. Care* **30**, 473–483 (1992).
7. Löwe, B. *et al.* Validation and standardization of the Generalized Anxiety Disorder Screener (GAD-7) in the general population. *Med. Care* **46**, 266–274 (2008).
8. Lee, E.-H. Review of the psychometric evidence of the perceived stress scale. *Asian Nurs. Res.* **6**, 121–127 (2012).
9. Hauksdóttir, A., McClure, C., Jonsson, S. H., Olafsson, O. & Valdimarsdóttir, U. A. Increased stress among women following an economic collapse--a prospective cohort study. *Am. J. Epidemiol.* **177**, 979–988 (2013).
10. Hlodversdottir, H., Petursdottir, G., Carlsen, H. K., Gislason, T. & Hauksdottir, A. Long-term health effects of the Eyjafjallajökull volcanic eruption: a prospective cohort study in 2010 and 2013. *BMJ Open* **6**, e011444 (2016).

11. Diener, E., Emmons, R. A., Larsen, R. J. & Griffin, S. The Satisfaction With Life Scale. *J. Pers. Assess.* **49**, 71–75 (1985).

12. Pavot, W. & Diener, E. Review of the Satisfaction With Life Scale. **5**, 164–172 (1993).

13. Ware, J. E. SF-36 health survey update. *Spine* **25**, 3130–3139 (2000).

14. Garratt, A. M., Ruta, D. A., Abdalla, M. I., Buckingham, J. K. & Russell, I. T. The SF36 health survey questionnaire: an outcome measure suitable for routine use within the NHS? *BMJ* **306**, 1440–1444 (1993).

15. Bullinger, M. *et al.* Translating health status questionnaires and evaluating their quality: the IQOLA Project approach. International Quality of Life Assessment. *J. Clin. Epidemiol.* **51**, 913–923 (1998).

16. Farivar, S. S., Cunningham, W. E. & Hays, R. D. Correlated physical and mental health summary scores for the SF-36 and SF-12 Health Survey, V.I. *Health Qual. Life Outcomes* **5**, 54 (2007).

17. Muntner, P. *et al.* Measurement of Blood Pressure in Humans: A Scientific Statement From the American Heart Association. *Hypertens. Dallas Tex 1979* **73**, e35–e66 (2019).

18. Roberts, H. C. *et al.* A review of the measurement of grip strength in clinical and epidemiological studies: towards a standardised approach. *Age Ageing* **40**, 423–429 (2011).

19. Gisladottir, R. S. *et al.* Sequence Variants in TAAR5 and Other Loci Affect Human Odor Perception and Naming. *Curr. Biol. CB* **30**, 4643-4653.e3 (2020).

20. Hummel, T., Sekinger, B., Wolf, S. R., Pauli, E. & Kobal, G. 'Sniffin' sticks': olfactory performance assessed by the combined testing of odor identification, odor discrimination and olfactory threshold. *Chem. Senses* **22**, 39–52 (1997).

21. Hummel, T., Kobal, G., Gudziol, H. & Mackay-Sim, A. Normative data for the 'Sniffin'' Sticks" including tests of odor identification, odor discrimination, and olfactory thresholds: an upgrade based on a group of more than 3,000 subjects'. *Eur. Arch. Oto-Rhino-Laryngol. Off. J. Eur. Fed. Oto-*

Rhino-Laryngol. Soc. EUFOS Affil. Ger. Soc. Oto-Rhino-Laryngol. - Head Neck Surg. **264**, 237–243 (2007).

22. Iravani, B. *et al.* Relationship between odor intensity estimates and COVID-19 prevalence prediction in a Swedish population. *Chem. Senses* (2020) doi:10.1093/chemse/bjaa034.

23. Vennemann, M. M., Hummel, T. & Berger, K. The association between smoking and smell and taste impairment in the general population. *J. Neurol.* **255**, 1121–1126 (2008).

24. Hummel, T., Hummel, C. & Welge-Luessen, A. *Assessment of olfaction and gustation. Management of smell and taste disorders: a practical guide for clinicians*. (Thieme, 2013).

25. Ivarsdottir, E. V. *et al.* The genetic architecture of age-related hearing impairment revealed by genome-wide association analysis. *Commun. Biol.* **4**, (2021).

26. Mathers, C., Smith, A. & Concha, M. Global burden of hearing loss in the year 2000. World Heal. Organ. 1–30 (2000). https://www.who.int/healthinfo/statistics/bod_hearingloss.pdf (2000).

27. Graham, B. L. *et al.* Standardization of Spirometry 2019 Update. An Official American Thoracic Society and European Respiratory Society Technical Statement. *Am. J. Respir. Crit. Care Med.* **200**, e70–e88 (2019).

28. Balady, G. J. *et al.* Clinician's Guide to cardiopulmonary exercise testing in adults: a scientific statement from the American Heart Association. *Circulation* **122**, 191–225 (2010).

29. Mezzani, A. *et al.* Standards for the use of cardiopulmonary exercise testing for the functional evaluation of cardiac patients: a report from the Exercise Physiology Section of the European Association for Cardiovascular Prevention and Rehabilitation. *Eur. J. Cardiovasc. Prev. Rehabil. Off. J. Eur. Soc. Cardiol. Work. Groups Epidemiol. Prev. Card. Rehabil. Exerc. Physiol.* **16**, 249–267 (2009).

30. Wasserman, K., Hansen, J., Sue, D., Whipp, B. & Casaburi, R. *Principles of exercise testing and interpretation*. (Lippincott Williams and Wilkins, 2004).

31. Ebaid, D., Crewther, S. G., MacCalman, K., Brown, A. & Crewther, D. P. Cognitive Processing Speed across the Lifespan: Beyond the Influence of Motor Speed. *Front. Aging Neurosci.* **9**, 62 (2017).

32. Stefansson, H. *et al.* CNVs conferring risk of autism or schizophrenia affect cognition in controls. *Nature* **505**, 361–366 (2014).

33. Ruff, R. M., Light, R. H., Parker, S. B. & Levin, H. S. Benton Controlled Oral Word Association Test: reliability and updated norms. *Arch. Clin. Neuropsychol. Off. J. Natl. Acad. Neuropsychol.* **11**, 329–338 (1996).

34. Morris, J. C. *et al.* The Consortium to Establish a Registry for Alzheimer's Disease (CERAD). Part I. Clinical and neuropsychological assessment of Alzheimer's disease. *Neurology* **39**, 1159–1165 (1989).

35. Bell, B. D. WMS-III Logical Memory performance after a two-week delay in temporal lobe epilepsy and control groups. *J. Clin. Exp. Neuropsychol.* **28**, 1435–1443 (2006).

36. Owen, A. M., Downes, J. J., Sahakian, B. J., Polkey, C. E. & Robbins, T. W. Planning and spatial working memory following frontal lobe lesions in man. *Neuropsychologia* **28**, 1021–1034 (1990).

37. Reitan, R. M. Validity of the trail making test as an indicator of organic brain damage. 271–276 (1958).

38. Wechsler. *Wechsler Abbreviated Scale of Intelligence (WASI) Manual. Psychological Corporation.* (1999).

39. Gudmundsson, E. Mat á greind fullorðinna: WASI-IS [The Assessment of Intelligence in Adults: WASI-IS]. *Menntamalastofnun* (2015).

40. Orphanidou, C. *et al.* Signal-quality indices for the electrocardiogram and photoplethysmogram: derivation and applications to wireless monitoring. *IEEE J. Biomed. Health Inform.* **19**, 832–838 (2015).

41. Gudbjartsson, D. F. *et al.* Humoral Immune Response to SARS-CoV-2 in Iceland. *N. Engl. J. Med.* **383**, 1724–1734 (2020).

42. Olafsdottir, T. A. *et al.* Eighty-eight variants highlight the role of T cell regulation and airway remodeling in asthma pathogenesis. *Nat. Commun.* **11**, 393 (2020).

43. Sigurdardottir, L. G. *et al.* Data quality at the Icelandic Cancer Registry: comparability, validity, timeliness and completeness. *Acta Oncol. Stockh. Swed.* **51**, 880–889 (2012).

44. Helgadottir, A. *et al.* Variants with large effects on blood lipids and the role of cholesterol and triglycerides in coronary disease. *Nat. Genet.* **48**, 634–639 (2016).

45. Steinthorsdottir, V. *et al.* Identification of low-frequency and rare sequence variants associated with elevated or reduced risk of type 2 diabetes. *Nat. Genet.* **46**, 294–298 (2014).

46. Sveinbjornsson, G. *et al.* Rare mutations associating with serum creatinine and chronic kidney disease. *Hum. Mol. Genet.* **23**, 6935–6943 (2014).

47. Gudbjartsson, D. F. *et al.* Association of variants at UMOD with chronic kidney disease and kidney stones-role of age and comorbid diseases. *PLoS Genet.* **6**, e1001039 (2010).
