# Supplementary material for: Physical and cognitive impact following SARS-CoV-2 infection in a large population-based case-control study
Source: Commun Med (Lond). 2023 Jul 6;3:94. doi: 10.1038/s43856-023-00326-5 (PMC10325957; doi:10.1038/s43856-023-00326-5)
Supplement: Supplementary file 10 — Reporting Summary [file 43856_2023_326_MOESM10_ESM.pdf]

nature research

Corresponding author(s): Hilma Holm

Last updated by author(s): Jun 2, 2023

Reporting Summary

Nature Research wishes to improve the reproducibility of the work that we publish. This form provides structure for consistency and transparency in reporting. For further information on Nature Research policies, see our Editorial Policies and the Editorial Policy Checklist.

Statistics

For all statistical analyses, confirm that the following items are present in the figure legend, table legend, main text, or Methods section.

n/a	Confirmed	
☐	☒	The exact sample size (n) for each experimental group/condition, given as a discrete number and unit of measurement
☒	☐	A statement on whether measurements were taken from distinct samples or whether the same sample was measured repeatedly
☐	☒	The statistical test(s) used AND whether they are one- or two-sided<br>*Only common tests should be described solely by name; describe more complex techniques in the Methods section.*
☐	☒	A description of all covariates tested
☐	☒	A description of any assumptions or corrections, such as tests of normality and adjustment for multiple comparisons
☐	☒	A full description of the statistical parameters including central tendency (e.g. means) or other basic estimates (e.g. regression coefficient) AND variation (e.g. standard deviation) or associated estimates of uncertainty (e.g. confidence intervals)
☐	☒	For null hypothesis testing, the test statistic (e.g. F, t, r) with confidence intervals, effect sizes, degrees of freedom and P value noted<br>*Give P values as exact values whenever suitable.*
☒	☐	For Bayesian analysis, information on the choice of priors and Markov chain Monte Carlo settings
☐	☒	For hierarchical and complex designs, identification of the appropriate level for tests and full reporting of outcomes
☐	☒	Estimates of effect sizes (e.g. Cohen's d, Pearson's r), indicating how they were calculated

Our web collection on statistics for biologists contains articles on many of the points above.

Software and code

Policy information about availability of computer code

Data collection	Hardware and software used to produce phenotypic data in the deCODE Health Study is described in Online Methods and Supplementary Methods. No other software was used to collect data.
Data analysis	We performed statistical analyses in R, version 3.6.0.

For manuscripts utilizing custom algorithms or software that are central to the research but not yet described in published literature, software must be made available to editors and reviewers. We strongly encourage code deposition in a community repository (e.g. GitHub). See the Nature Research guidelines for submitting code & software for further information.

Data

Policy information about availability of data

All manuscripts must include a data availability statement. This statement should provide the following information, where applicable:

- Accession codes, unique identifiers, or web links for publicly available datasets
- A list of figures that have associated raw data
- A description of any restrictions on data availability

Individual participant data from the deCODE Health Study will not be made available to others. The study protocol and statistical analysis plan are described in this paper and more detailed information is available on request.

Field-specific reporting

Please select the one below that is the best fit for your research. If you are not sure, read the appropriate sections before making your selection.

☒ Life sciences ☐ Behavioural & social sciences ☐ Ecological, evolutionary & environmental sciences

For a reference copy of the document with all sections, see nature.com/documents/nr-reporting-summary-flat.pdf

Life sciences study design

All studies must disclose on these points even when the disclosure is negative.

Sample size	We used all data available to us in the deCODE Health Study to perform this research that is, derived from the 13,779 persons that participated in the study before the pandemic (historical controls) and the 1,706 persons that had contracted SARS-CoV-2 (confirmed cases) and 619 contemporary controls that participated in the study between September 2020 and November 2021. We did not perform any particular power calculations prior to the initiation of the study.
Data exclusions	We have analyzed most but not all data collected for participants in the study. Data yet to be analyzed are mainly phentypic data that require processing that has not yet been performed, such as in depth analysis of sleep data, and OCT retinal images.
Replication	We have not tried to replicate our findings. This will require another large phenotypic study.
Randomization	Study participants were not randomized but allocated into cases/controls depending on prior SARS-CoV-2 infection. The majority of cases had been diagnosed with infection by PCR and all infections were confirmed by antibody measurements. The absence of prior infection among controls was also confirmed by antibody measurements.
Blinding	The investigators were not blinded to history of prior infections. This was not possible give the nature of the study.

Reporting for specific materials, systems and methods

We require information from authors about some types of materials, experimental systems and methods used in many studies. Here, indicate whether each material, system or method listed is relevant to your study. If you are not sure if a list item applies to your research, read the appropriate section before selecting a response.

Materials & experimental systems

n/a	Involved in the study	
☒	☐	Antibodies
☒	☐	Eukaryotic cell lines
☒	☐	Palaeontology and archaeology
☒	☐	Animals and other organisms
☐	☒	Human research participants
☒	☐	Clinical data
☒	☐	Dual use research of concern

Methods

n/a	Involved in the study	
☒	☐	ChIP-seq
☒	☐	Flow cytometry
☒	☐	MRI-based neuroimaging

Human research participants

Policy information about studies involving human research participants

Population characteristics	For this study we adjusted the deCODE Health Study protocol to address specific factors of interest in relation to a prior infection with SARS-CoV-2. We invited to the dHS Covid study Icelanders that had been diagnosed with SARS-CoV-2 at least five months prior to the study initiation as well as age and sex matched Icelandic controls. 50% of the cases that participated were women, 50% of the contemporary controls were women, 57% of historical controls were women.
Recruitment	We invited to the dHS Covid study Icelanders that had been diagnosed with SARS-CoV-2 at least five months prior to the study initiation as well as age and sex matched Icelandic controls (contemporary controls). It is possible that cases (those with prior infection) that were particularly concerned about their health after the infection were more likely to participate in the study. It is also possible that those that were the sickest during the acute infection were less likely to participate as the participation was a lengthy (4 hour) process. However, demographics and co morbidities were similar among those who participated and those who did not, and 5% of the cases had required hospitalization which is the same fraction as noted overall for hospitalization for SARS-CoV-2 infected in Iceland. In general it is possible that individuals with the poorest health did not participated in the study, but that would be expected to apply equally to cases and controls.
Ethics oversight	Written informed consent was obtained from all participants, in accordance with the Declaration of Helsinki, and the study was approved by the Icelandic National Bioethics Committee (VSN-15-214 with amendments).

Note that full information on the approval of the study protocol must also be provided in the manuscript.
